# Sport Events for Sport Participation: A Scoping Review

**DOI:** 10.3389/fspor.2021.655579

**Published:** 2021-05-19

**Authors:** Georgia Teare, Marijke Taks

**Affiliations:** Faculty of Health Sciences, School of Human Kinetics, University of Ottawa, Ottawa, ON, Canada

**Keywords:** legacy, leverage, impact, small scale sport events, mega sport events

## Abstract

Research on sport participation impacts from sport events has been sporadic. This scoping review assesses the current state of literature that addresses impacts, legacies, and leveraging of sport events for sport participation outcomes and the gaps in terms of study context and research designs. Two systematic approaches of article identification were performed: a traditional database search and a systematic manual search. Studies on sport participation outcomes from events mainly focused on mega sport events and adult populations, with the majority employing cross-sectional data and quantitative methods. The use theoretical of frameworks is limited and inconsistent. There is a need for longitudinal investigations, as well as more focus on youth populations, participant events, and smaller-sized events to advance the research agenda for sport participation outcomes from sport events.

## Introduction

Prior to 2005, the thought that sport participation is inherently associated with sport events remained largely anecdotal, with little academic attention given to the phenomenon (Coalter, 2004; Weed et al., 2015). The scant empirical research into this notion had in fact not supported the idea that elite athletic success trickles down into grassroots participation (e.g., Hindson et al., 1994). In 2005, however, the London 2012 bid committee submitted a bid to host the 2012 Olympic Games with the explicit legacy claim of inspiring a generation to be more physically active (Weed et al., 2015). As such, academic researchers took notice, and sport participation as a possible legacy of sport events has become an increasingly popular line of inquiry (e.g., Annear et al., 2019), including in the field of sport management (Weed et al., 2015). Indeed, impacts (i.e., changes directly associated with an event), legacies (i.e., long-term impacts; planned or unplanned), and leveraging (i.e., strategically planned impacts) of sport events are starting to receive more attention in sport management scholarship as it focuses on impact assessment, process-oriented management, optimization of event outcomes to inform sport, and event planning and management (Taks et al., 2015).

To date, there is little empirical support for the inherent association between staging sport events and sport participation (Weed et al., 2015; Annear et al., 2019). Such findings have led to the notion that explicit plans must be made prior to the event to achieve desired outcomes, and the plans must be managed during the lead-up, implementation of, and after the event takes place (Chalip et al., 2017). This process is referred to as leveraging (Chalip, 2006). Event leveraging involves using an event to elicit further indirect benefits outside the event's primary goal that would have occurred at a much slower rate had the event not taken place (Chalip, 2006, 2014). However, research on the association between hosting sport events and sport participation and leveraging sport events for participation outcomes has been sporadic, and systematic types of reviews conducted on the phenomenon have mainly focused on mega-spectator sport events (Weed et al., 2015), generally neglecting smaller-sized spectator and participant events. Indeed, there is a need for a more comprehensive overview including a greater variety of contexts and events to illustrate and synthesize these findings from a broader perspective. Thus, the purpose of this study is to conduct a scoping review to assess the current state of literature that addresses impacts (i.e., changes directly associated with an event), legacies (i.e., long-term impacts; planned or unplanned), and leveraging (i.e., strategically planned impacts) of sport events for sport participation outcomes and the gaps in terms of study context and research designs. The terms impact, legacy, and leverage are indeed all related to changes associated with events. They are all different, however, with regard to strategic and structural considerations. Impacts, though not formally defined in the literature, involve any change associated with the event. These could be changes that are planned or unplanned, within the host community or outside of it, and can take place at any point temporally. Legacy and leverage have more formal definitions and conceptualizations. Legacy is the long-term impact of an event that can be planned or unplanned, positive or negative, tangible or intangible, which remains in the host community long after the event is over (Preuss, 2019). Leveraging, however, is a strategic approach to using a sport event to achieve desired outcomes (Chalip, 2006).

Outcomes that have been posited to date, and how we define sport participation from sport events, include those who currently participate in sport increase their participation rates; current participants switching or diversifying sports; former participants starting to participate again; and the most contentious form of participation being nonparticipants taking up sport for the first time (e.g., Weed et al., 2015). In other words, we are interested in the change in nature of sport participation. These outcomes may come about at any stage of the event—in event lead-up, prior to the event taking place; during the event; or post-event.

Indeed, scholars have attempted to synthesize findings of research conducted on the phenomenon of sport participation from sport events to come to conclusions about the ability of sport events to stimulate sport participation (McCartney et al., 2010; Weed et al., 2015; Annear et al., 2019; Thompson et al., 2019; Thomson et al., 2020; Potwarka and Wicker, 2021). Such reviews, however, have taken focused approaches to evaluate specific types of events or contexts. For instance, the systematic review by Weed et al. (2015) investigated sport participation impacts of the Olympic and Paralympic Games. Similarly, McCartney et al. (2010) investigated health and socioeconomic impacts of major multi-sport events through a systematic review, and Annear et al. (2019) conducted a systematic review of sport participation impacts of mega sport events. However, the review by Annear et al. (2019) only included studies that addressed adult populations. Potwarka and Wicker (2021) considered empirical studies that specifically addressed the trickle-down effect (i.e., spectators inspired by athletes to participate in sport or physical activity). Finally, two systematic quantitative literature reviews by Thomson et al. (2020), Thomson et al. (2020) explicitly focused on legacy and spectator sport events, neglecting research investigating non-legacy impacts (e.g., short-term impacts, leveraging) and participant-based events. Five of the six reviews (e.g., McCartney et al., 2010; Weed et al., 2015; Annear et al., 2019; Thompson et al., 2019; Thomson et al., 2020) found no inherent effect on sport participation and/or physical activity associated with hosting major sport events, while the remaining review identified specific conditions under which the specific phenomenon of the trickle-down effect might occur (e.g., Potwarka and Wicker, 2021). The targeted scope of these reviews creates boundaries to studying sport participation from sport events. A wider variety of different contexts in terms of event size (i.e., size, scale, and scope; small, medium, large, mega; multi-sport to single sport, etc.), type of event (participant/spectator), and study population (e.g., youth/adults) should also be considered when undertaking future investigations. By broadening the scope of inclusion for a review of the literature on sport participation from sport events, we can draw attention to different contexts and study designs for investigating the phenomenon. Thus, a more comprehensive review of literature on sport participation impacts of various sizes and types of sport events will add to this body of knowledge. Therefore, this scoping review provides an overview of a wide variety of studies examining the relationship between sport events and sport participation outcomes. Their various study contexts (i.e., size of events, type of event, study population) and associated research designs (i.e., theory, study design, methodology, type of data, methods) are examined. Possible gaps in study context and research designs can then be determined, which can subsequently inform avenues for future research.

To achieve a comprehensive review of the literature, two systematic approaches of article identification were performed—a traditional database and a systematic manual search. This dual approach is based on previous findings that both methods lead to different pools of identified articles; one method of article identification can omit relevant articles that can be identified through other strategies (Teare and Taks, 2020). As such, we first present the methodological approach for the execution of the scoping review. Next, the current status of research in this domain is presented, and avenues for future research are identified and discussed.

## Method

Arksey and O'Malley (2005) identified four distinct purposes for conducting a scoping review that fall under two broader ways of thinking. The first way of thinking is considering the scoping review as a preliminary step to a systematic review. The possible purposes associated with this way of thinking would be (1) to map the fields of study to assess the range of evidence and (2) to assess if conducting a systematic review is necessary (Arksey and O'Malley, 2005). The second broad way of thinking is to consider the scoping review as a stand-alone study. An associated purpose would be (3) to summarize and disseminate findings for practitioners (Arksey and O'Malley, 2005). The final purpose, and the one undertaken in the current study, is (4) to identify gaps in the existing literature by assessing the overall state of research evidence (Arksey and O'Malley, 2005).

A scoping review, which is by default systematic, assesses the nature and extent of research evidence in a replicable and rigorous way (Grant and Booth, 2009; Whittemore et al., 2014). A scoping review provides an overview of a particular line in inquiry, including the size of available literature, scope of studies, and highlights gaps in methodologies and findings (Grant and Booth, 2009). Unlike other types of systematic approaches to reviews, such as systematic reviews or meta-analyses, a scoping review does not appraise or synthesize the findings of the articles, as was the case for the systematic reviews on sport participation from sport events previously mentioned (i.e., McCartney et al., 2010; Weed et al., 2015; Annear et al., 2019). The articles are not assessed within the parameters of a scoping review to avoid exclusion of relevant literature. As the phenomenon examined here is a relatively new line of inquiry, all investigations (i.e., both empirical and conceptual peer-reviewed articles) should be considered to add to the understanding of the current state of literature. Thus, the purpose of the current review is to understand the state of the literature rather than provide a synthesis of findings regarding the ability of events to stimulate sport participation.

The scoping review was executed following the five-step framework (Table 1) of Arksey and O'Malley (2005) and was extended with a comprehensive systematic manual journal search framework following the guidelines of Teare and Taks (2020). The selection process was established based on the following research question: “What is the current state of literature that addresses impacts, legacies, and leveraging of sport events for sport participation outcomes in terms of study context and research designs?” (step 1).

**Table 1:** Scoping review framework (Adapted from Arksey and O'Malley, 2005).

**Step**	**Description**
1. Identifying the research question	Guide the type of review and the parameters of the review
2. Identifying relevant studies	Help determine what sources to search, appropriate search terms, time span, and language. Potential sources include electronic databases, reference lists, hand searching of key journals, and organizations and conferences
	Minimum of two systematic approaches such as database search and systematic manual search
3. Study selection	Using inclusion and exclusion criteria, based on the research question, to search the sources chosen
4. Charting the data	Data-charting form is developed and used to extract data from each study
5. Collating, summarizing, and reporting results	Analytic framework or thematic construction is used to provide an overview of the breadth of the literature but not a synthesis. A numerical analysis of the extent and nature of studies using tables and charts is presented. A thematic analysis is then presented

*Gray background indicates extension of the framework of Arksey and O'Malley (2005) and Teare and Taks (2020)*.

### Article Selection Process

Preliminary readings of related articles (e.g., Veal et al., 2012; Weed et al., 2015; Chalip et al., 2017; Taks et al., 2018) served to identify key words and terms to select the articles (step 2). The following inclusion criteria were determined: peer-reviewed articles or book chapters; available online; written in English; address impacts, legacies, leveraging of sport events; with sport participation as an outcome, not as a potential application. For example, studies that measured sport participation rates or assessed sport participation experiences were included, while studies that empirically assessed outcomes of sport events that are not sport participation (e.g., livability of areas on London; Smith et al., 2015) but mentioned potential sport participation implications only were excluded. The database search took place in January 2019, including articles published up to and including 2018, and the comprehensive systematic manual search included all issues up to and including 2018.

#### Database Search

Databases were chosen based on the research question and their likelihood to contain relevant articles. Based on preliminary readings, and in consultation with a research librarian at the author's institution, the following five databases and key words were used to search for articles published up to and including 2018: ABI Inform, Business Source Complete, Physical Education Index, SPORTDiscus, and Web of Science. The following key search words were used “sport^*^ N2 participat^*^” OR “physical^*^ N2 activ^*^” AND “sport^*^ event^*^” OR Olympic^*^ OR championship^*^ OR “FIFA” OR “World Cup.” This initial search revealed 3,013 total articles. Duplicates were then removed, leaving 2,252 articles to be searched in the first round of screening. By keeping the search very broad and not including specific terms related to the nature of the sport participation impact (i.e., impact, legacy, or leverage), we were able to capture a wider range of articles.

**Figure 1:**
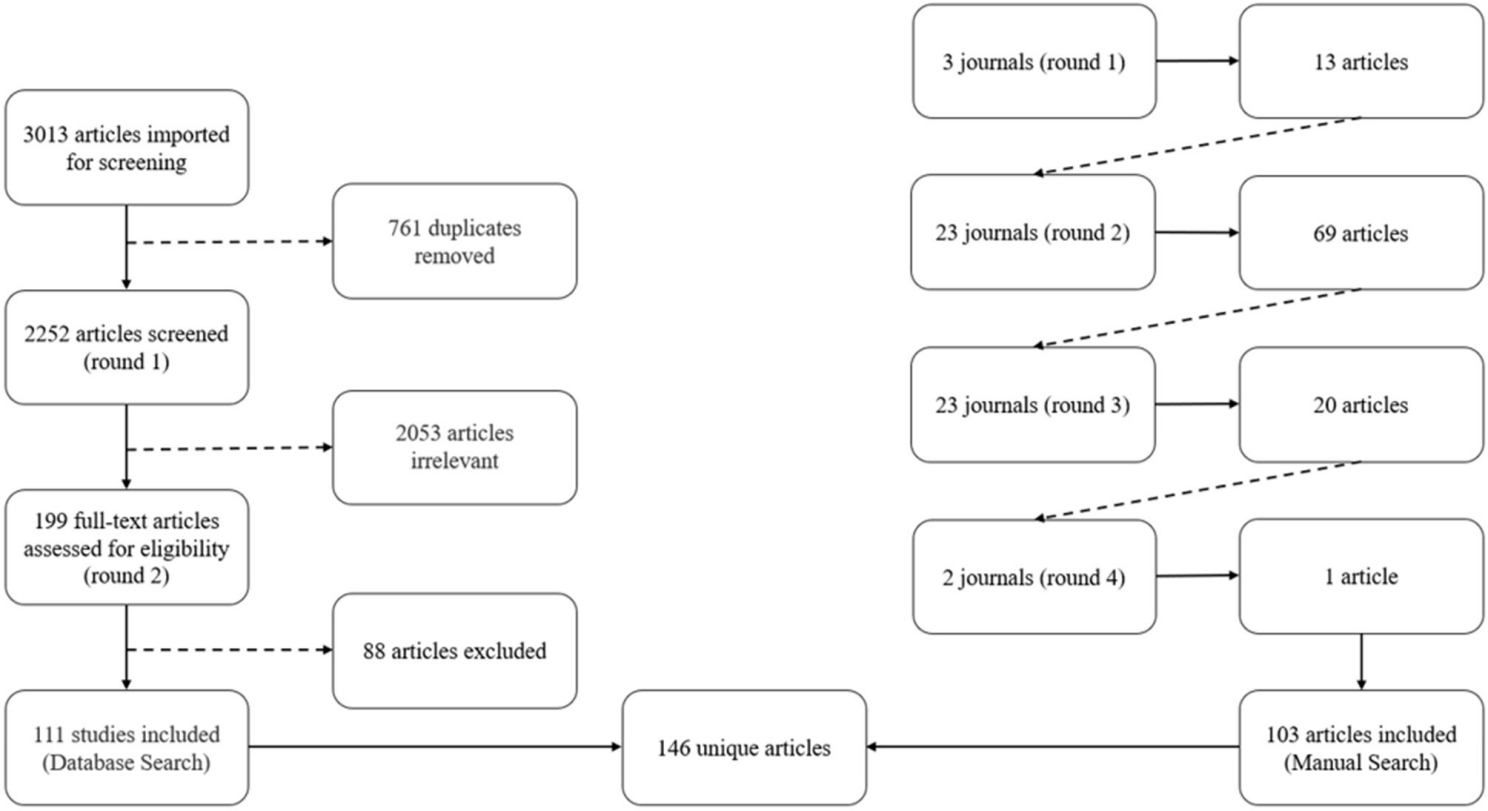
Flow diagrams for the traditional database search and systematic manual search.

The database search involved two rounds of screening to determine if the articles were to be included in the final pool of sources (step 3). Two researchers screened the articles independently. First, titles and abstracts were screened against the inclusion and exclusion criteria. Articles that were deemed to fit with the inclusion criteria by both researchers were moved directly to the second round of full-text screening. Articles that were excluded by both researchers were immediately eliminated. When the researchers disagreed, the articles were flagged, and both researchers met to discuss the titles and abstracts against the inclusion/exclusion criteria. Articles that were mutually agreed to be potentially relevant were moved to the second round of screening. After this first round of screening (title and abstracts) and discussing discrepancies, 2,053 articles were removed, and 199 articles were moved onto the second round of screening (full text). The authors then separately read all the articles of this second round in full. Articles that were agreed upon were included in the final pool of articles, and discrepancies were again discussed. Of essence was that a focus on sport participation outcomes had to be presented. After the second round of full-text screening, the database search yielded 111 relevant articles from five databases. Similar to McCartney et al. (2010) and Weed et al. (2015), we present the article selection process of the database search visually in Figure 1 (the left-hand side).

#### Systematic Manual Search

As per Teare and Taks (2020), there are three steps involved in conducting a systematic manual search: (1) selecting the top field-specific journals (as determined by impact factors) within the specified time frame to begin the search, (2) screening all issues for relevant articles (i.e., two rounds of screening of titles and abstracts, followed by full texts), and (3) examining the reference lists of the identified articles for additional relevant journals. The same process is then completed for the identified journals, and a full journal search of these new journals is performed until no new journals arise (Teare and Taks, 2020).

In the context of this study, the relationship between sport participation and events is examined through the lens of impacts, legacies, and leveraging. Though these themes can be found in other domains, through preliminary reading of articles, it was identified that sport participation from sport events is substantially researched in the field of sport management. Thus, sport management journals were a relevant starting point to serve the research purpose and question. It is important to note that though sport management journals serve as a starting point for the systematic manual search, the search is not limited to only sport management journals. In fact, as identified by Teare and Taks (2020), the systematic manual search is particularly useful when conducting scoping reviews on interdisciplinary topics such as the one in the present study, as it is able to identify journals and articles from several different domains. Teare and Taks (2020) suggest that this could be due to the varying terminology used in different domains to investigate a similar topic that might not be captured by the key words used in a database search.

Looking at impact factors, the top 3 journals in sport management are *Sport Management Review, Journal of Sport Management*, and *European Sport Management Quarterly*. All articles in all issues were examined using the inclusion and exclusion criteria. Titles, abstracts, and key words comprised the first round of screening, followed by full-text screening. The top 3 journals generated 13 articles. As indicated above, the reference lists of the identified articles were examined for additional journals, revealing 23 journals to be reviewed in round 2. The 23 journals of round 2 yielded 69 relevant articles whose reference lists identified an additional 23 journals. These 23 journals of round 3 were again fully searched, generating 20 relevant articles. These articles revealed an additional two journals in their reference list for a full search in round 4. The final two journals yielded one additional article. No new journals appeared in the article's reference list. Thus, four rounds of journal searches were undertaken until no new journals arose. In total, the systematic manual search yielded 51 journals (3 + 23 + 23 + 2 = 51) for a total 103 articles (13 + 69 + 20+ 1 = 103). The process of the systematic manual search is visually represented in Figure 1 (right-hand side).

#### Combining the Search Methods

The database search yielded 111 relevant articles, while the systematic manual search yielded 103 relevant articles, for a total of 214 total identified articles. When combining the results of the two search methods, only 68 articles were identified through both searches. Thus, this scoping review included 146 total unique articles (43 articles unique to the database search; 35 articles unique to the systematic manual search; 68 articles identified through both search methods). All articles included in the scoping review as well as their source search method are available online as a Supplementary File. The distinctive outcome between the database search and the manual search is important, as it informs researchers that one or the other method alone is insufficient to generate a comprehensive overview of the work in a specific area. Teare and Taks (2020) suggested that for interdisciplinary topics such as the phenomenon being addressed by the current study, more than one rigorous search strategy must be employed when conducting a comprehensive review. The need for multiple rigorous search strategies may stem from the different terminologies used in different fields to describe the same phenomenon (Teare and Taks, 2020). This notion is confirmed by the systematic literature review by Geurin and Naraine (2020) who adopted the approach of Teare and Taks (2020) to article identification. The database search by Geurin and Naraine (2020) yielded 173 unique articles, and their systematic manual search yielded an additional 48 unique articles that did not appear in the database search.

### Data Analysis

Covidence (www.covidence.org), a systematic review management tool, was used to streamline the study selection process (systematic review as an umbrella term for systematic approach to conducting a review; Grant and Booth, 2009). All relevant articles were imported into the system, allowing the researchers to analyze the articles independently from one another. Categories of information to extract from the articles (i.e., step 4 in the scoping review framework; Arksey and O'Malley, 2005) were developed prior to extracting the data based on preliminary reading of systematic and scoping reviews (e.g., Allender et al., 2006; Filo et al., 2015; Dowling et al., 2018). In addition to basic journal-identifying data [i.e., author(s), year of publication, journal, article title, key words], particular attention was given to the study context. Unlike previous reviews of literature on sport participation from sport events (e.g., McCartney et al., 2010; Weed et al., 2015; Annear et al., 2019), the present review considers events that vary in size and scope (event size) and takes into account the type of event based on the way in which participants engage with the event (type of event: spectator or participant event) and (3) various populations (study population). This broader and more comprehensive approach highlights several key trends and identifies which research contexts are lacking, allowing to investigate the effectiveness of different sizes and types of events in stimulating sport participation, with particular consideration given to specific study populations. For the purpose of this scoping review, we used sport event typologies of Müller (2015) and Gammon (2012) to distinguish between different sizes of events. Olympic Games and the FIFA world cup constitute mega sport events (Müller, 2015). All other elite international championship events (single or multisport) are considered large events. Small and medium events include charity and community events, non-elite international events, and elite national competitions (single sport). As many scholars did not state the scale of these community and non-elite events, small- and medium-sized events were considered to be the same category (Gammon, 2012).

Study context was followed by the elements of the research design. An overview of the data extraction criteria is provided in Table 2.

**Table 2:** Data extraction categories.

**Type of data extracted**	**Description**
**General information**	
Authors	The research team
Year of publication	The year the article was published
Journal	The journal in which the article was published
Purpose	Study's main purpose, research questions, and objectives
**Study context**	
Event	Name of the event(s) under investigation in the study
Event size	Mega (for the purpose of this study, summer Olympic Games, and FIFA), large, medium, small
Type of event	Spectator or participant event
Study population	Youth, adults, families, population-level, organizations, etc.
**Research design**	
Theory	If the study explicitly stated a theoretical framework or theoretical approach, and if so, which one(s)
Study design	Longitudinal, cross-sectional, time-series, Case study, PAR, etc.
Methods	Study methods (quantitative, qualitative, mixed methods)
Type of data	Primary, secondary, or both
Data collection instruments	Specific instruments employed for data collection (documents, surveys, interviews, etc.)
Research findings	If the study found support for the event under investigation to impact sport participation (i.e., yes, no, mixed)

## Results and Discussion

This section reflects step 5 of the framework by Arksey and O'Malley (2005): reporting and summarizing the results. The following sections report and summarize the frequency of publications around sport participation from sport events, the study contexts of these publications, the research designs used, and the research findings of these studies.

### Publication Frequency

**Figure 2:**
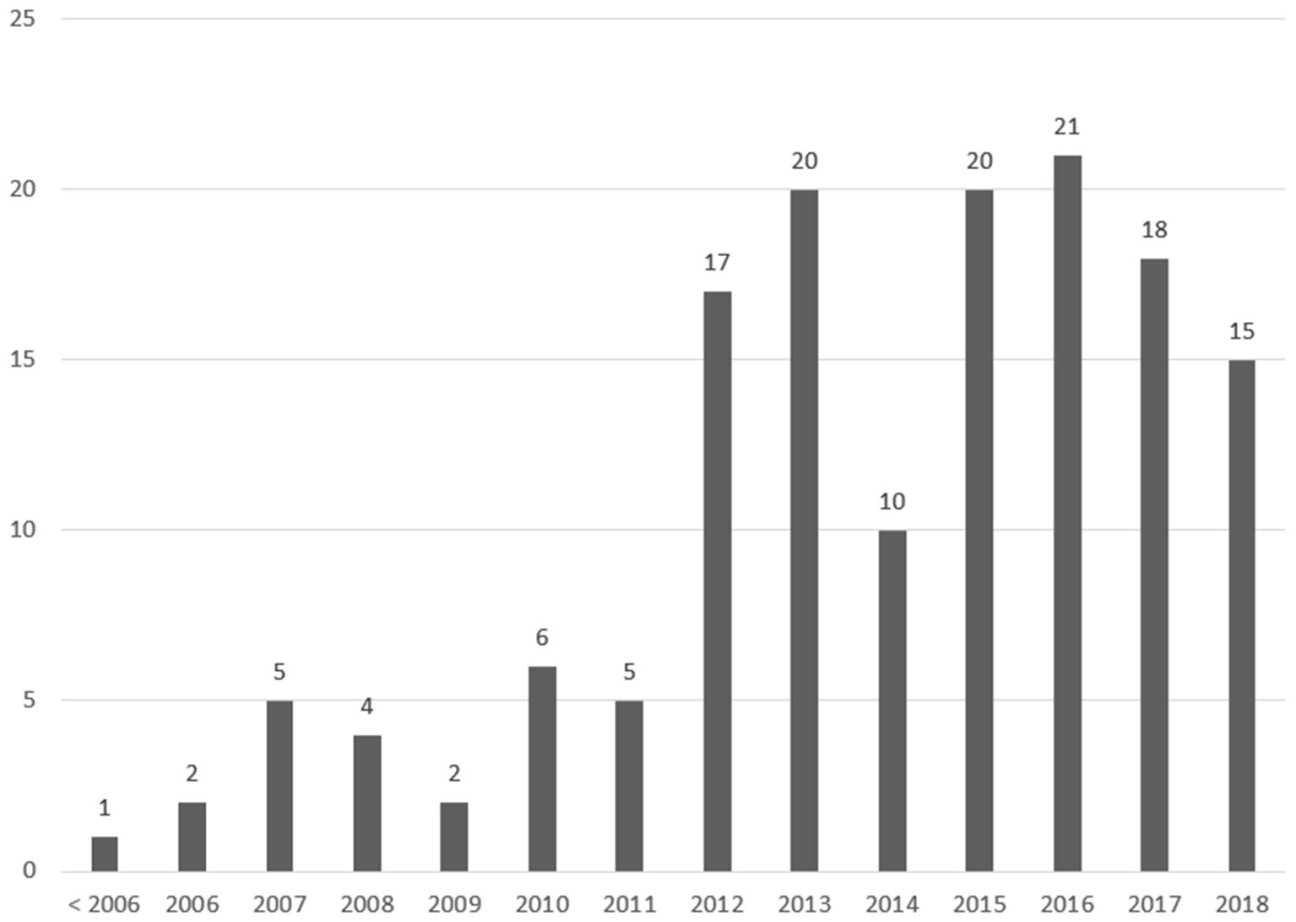
Year of publication.

There has been an increasing trend of articles published on the topic of sport participation from sport events. As seen in Figure 2, there is a larger increase in the number of articles published around the 2012 Olympic Games. This spike makes historical sense, as the 2012 Olympic Games were the first major sport event to explicitly claim, and plan for, a mass sport participation legacy (Weed et al., 2015). Many of the articles published in 2010, 2011, and 2012 were prospective articles, commenting on and examining the potential and likelihood of the London Games on achieving the participation goals (e.g., Charlton, 2010; Wellings et al., 2011; Bloyce and Lovett, 2012; Bullough, 2012; Hughes, 2012; Selvanayagam et al., 2012). Many of the articles published on the 2012 Olympic Games from 2015 onward were evaluations of the 2012 Games' impact on sport participation in London and the United Kingdom (e.g., Black et al., 2015; Mackintosh et al., 2015; Chen and Henry, 2016; Darko and Mackintosh, 2016; Girginov, 2016; Brown et al., 2017; Hayday et al., 2017; Lovett and Bloyce, 2017; Brown and Pappous, 2018).

### Study Context

#### Event Size

**Figure 3:**
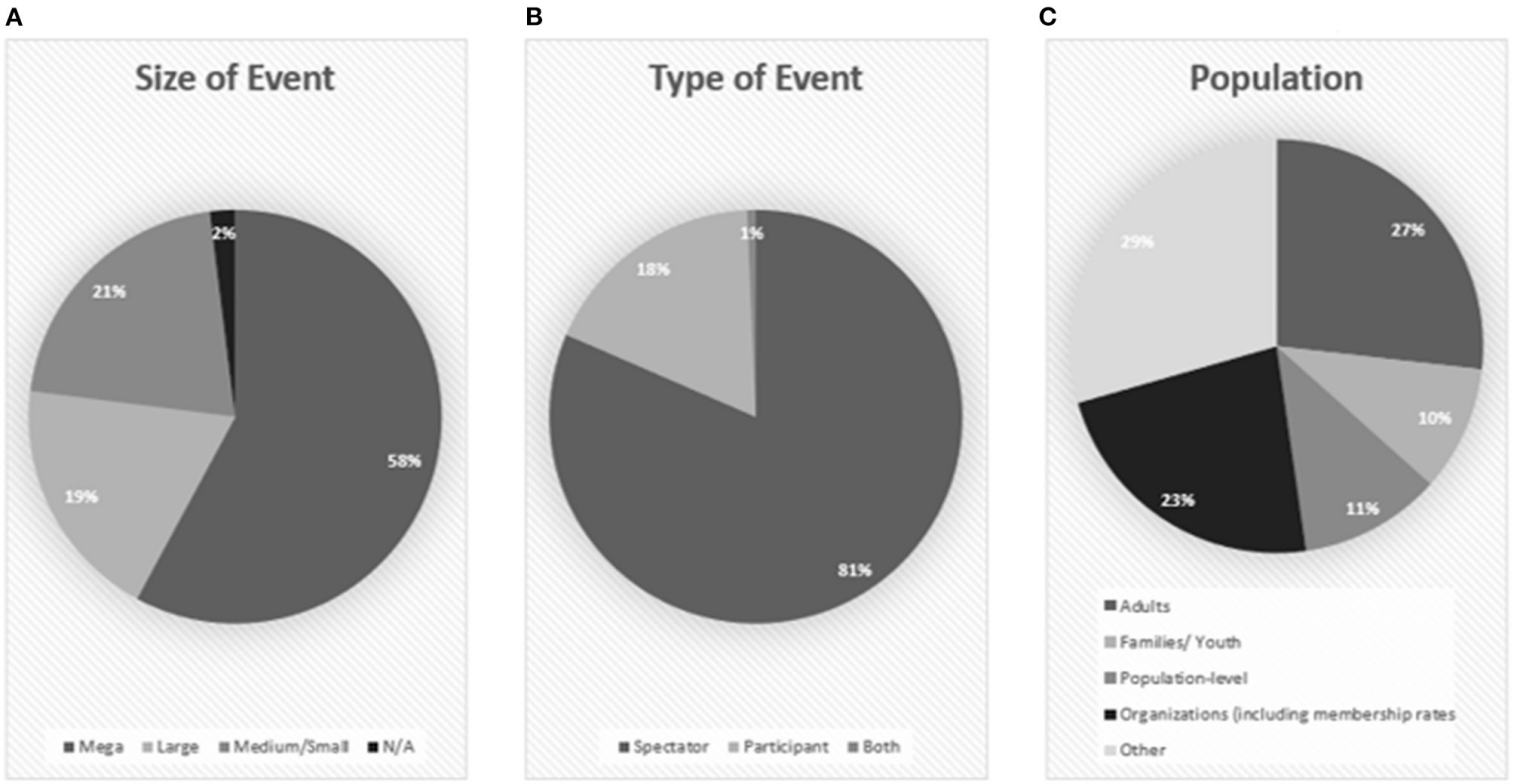
Study context: Size of event **(A)**, Type of event **(B)**, and Study population **(C)**.

As seen in Figure 3A, mega (*n* = 88; 58%) and large (*n* = 29; 19%) events combined account for over three-quarters of the studies included in this scoping review. Only 21% (*n* = 32) of the studies consider the impacts of small- and medium-sized sport events on sport participation, indicating that more research in this context is encouraged. The 2% (*n* = 1) of articles that were not applicable discussed sport events in general, not commenting on particular size of events (i.e., Inoue et al., 2015). In alignment with the year of publication trends corresponding to the 2012 Olympic Games, 52 (36%) studies included in this scoping review addressed the London 2012 Olympic Games. Other events that were commonly addressed include other Olympic Games (*n* = 41; 28%), the Commonwealth Games (*n* = 9; 6%), world or regional international championships (e.g., FIFA, World Rugby Championships, Pan American Games) (*n* = 22; 15%), and qualifying or community events (e.g., participant running events, elite tennis events) (*n* = 23; 16%).

A noteworthy finding with regard to the event size studied is that major multisport events tend to be studied without the inclusion of their parasport counterparts and *vice versa*. In other words, events like the Olympic Games or PanAmerican Games are studied without the inclusion of the Paralympic and Para-PanAmerican Games context, and the Paralympics and Para-PanAmerican Games are studied without the inclusion of the Olympic and PanAmerican Games context. For some investigations, keeping these events separate makes sense. For example, Coates and Vickerman (2016) considered the impact of the 2012 Paralympics for children with disabilities. Other investigations, however, could benefit from considering both the able-bodied and para iterations of events together, as discussed below, some populations have been found to be discouraged from sport participation by viewing elite able-bodied athletes but may be inspired by para-athletes (Carter and Lorenc, 2015). Future research on sport participation from major multisport events with able-bodied and parasport iterations should consider investigating both event contexts to better understand the overall impact of the event on the host community.

#### Type of Event

A similar trend appears with regard to type of event (i.e., spectator or participant) in the event. Here, 81% (*n* = 119) of the studies address spectator events, while 18% (*n* = 26) address participant events, and 1% (*n* = 1) address both spectator and participant events (Figure 3B). This trend indicates that there is still much to be researched with regard to the power of participant events to impact sport participation. As discussed below, participant events can attract nonparticipants to take up sport (training for the event) and are associated with post-event continued participation (Bowles et al., 2006; Crofts et al., 2012). If a goal of an event is to stimulate sport participation, perhaps those in decision-making positions should consider hosting participant-based events rather than spectator events. Alternatively, the nature of participant events to attract new participants can be strategically leveraged with regard to participant events (e.g., hold a participant event alongside a spectator event; Derom et al., 2015) to optimize attraction of nonparticipants.

#### Study Population

As seen in Figure 3C, many of the studies included in this scoping review examined adult's sport participation practices (*n* = 41; 27%) or examined the sport organization's perspective (*n* = 35; 23%) on the ability of sport events to stimulate sport participation. Few studies considered population-level changes in overall sport participation (*n* = 17; 11%) or youth populations (*n* = 15; 10%). The limited focus on youth is particularly problematic as many of these claims of increases in sport participation are made about youth populations. Moreover, the majority of studies that include youth populations are conducted in the context of mega or large sport events, such as the London Olympics (e.g., Griffiths and Armour, 2013; Mackintosh et al., 2015; Darko and Mackintosh, 2016; Kohe and Bowen-Jones, 2016; Sandercock et al., 2016; Such, 2016; Kohe, 2017) and Vancouver Olympics (e.g., Craig and Bauman, 2014; Potwarka and Leatherdale, 2016). Of note, Dubnewick et al. (2018) and Silvey et al. (2018) considered the impact of the Indigenous Traditional Games and the Newfoundland and Labrador Winter Games, respectively, on Indigenous youths' sport participation. Both Games investigated were in fact small- or medium-scale participant events. While participating in the Indigenous Traditional Games enhanced sport experiences and helped develop a foundation of movement for youth (Dubnewick et al., 2018), youth who had participated in the Newfoundland and Labrador Winter Games reported intentions to remain active after the Games. However, many participants did not attribute their participation intentions to participation in the Games, but rather they would have continued to participate in sport if they had not participated in the event (Silvey et al., 2018). Thus, future research should investigate how (if at all) sport events affect youths' sport participation.

Indeed, marginalized populations have received some attention by researchers in the literature thus far. In addition to the work by Dubnewick et al. (2018) and Silvey et al. (2018) with Indigenous youth described above, researchers have considered some marginalized populations, such as people with a disability (e.g., Misener, 2015; Misener et al., 2015a; Coates and Vickerman, 2016). There can, however, be a more target research agenda to strategically leverage sport events to provide sport participation opportunities for those who have traditionally been excluded from sport (e.g., low-income populations, homeless populations, LGBTQ+ populations, etc.).

### Research Design

#### Theory

In alignment with findings of Annear et al. (2019) on lack of theory used to investigate sport participation from sport events, well over half the studies included in this scoping review did not clearly identify a theoretical framework. Of the 54 studies that did state a guiding framework, the most common theoretical or guiding frameworks include Transtheoretical Model (*n* = 5), an Ecological Model (*n* = 4), and a realist evaluation (*n* = 3). The empirical articles employing the Transtheoretical model are all from the same research team (i.e., Ramchandani and Coleman, 2012; Ramchandani et al., 2015, 2017a,b). All four studies use surveys to assess adult elite sport event spectators' post-event intentions to participate in sport. All four studies also found that those who were inspired to intend to be active post-event were already active pre-event. The Transtheoretical model might be helpful in future investigations to understand behavior change associated with sport events; however, the contexts described here indicate that people who are already active engage with sport events. As discussed in sections below, research is needed to understand how events can be leveraged to engage inactive people with sport and physical activity.

Three studies (i.e., Derom and VanWynsberghe, 2015; Derom et al., 2015; Aizawa et al., 2018) employ a socioecological model, while one (i.e., Dubnewick et al., 2018) employs an Indigenous ecological model. These studies highlight the interconnectedness among individuals and event structures to suggest how sport events can impact community members. Social Ecology Theory (Stokols, 1992) suggests that individuals' behavior comes about from their interaction with both their physical (e.g., geography, architecture, technology) and sociocultural (e.g., culture, economics, politics) surroundings. The theory can be a useful tool for researchers to understand how leveraging initiatives can be designed to stimulate sport participation in a community and thus seems to have great potential in future work on sport event leveraging.

Finally, three studies employ a realist evaluation framework (i.e., Chen and Henry, 2016; Girginov, 2016; Bell and Daniels, 2018). These investigations place an emphasis on the context of the event and its ability to stimulate sport participation, particularly with regard to the resources provided for the purpose of increasing sport participation. A realist evaluation can be helpful in understanding how different types and sizes of events might be able to be leveraged by considering the event context and resources available. This approach might be helpful for researchers to consider when determining how different types and sizes of sport events might be able to be leveraged.

Other theories that were used by two studies include critical realism, neo-institutionalism, figuration theory, Foucault, Ansoff matrix, and Theory of Planned Behavior. There were an additional 29 other theories employed; however, each was only used in one study. The use of various theoretical lenses may be due to the diverse context of sport events to examine relevant sport-participating outcomes. On the other hand, the lack of theory use indicates that there is still little theoretical understanding of sport participation impacts of sport events. As such, future research might consider taking a grounded-theory approach to explore the phenomenon to develop a theory of sport participation from sport events.

It is also important to note that detailed models have been developed specifically to guide leveraging sport events for sport participation. Chalip et al. (2017) developed a model for leveraging sport events for sport participation and development. Taks et al. (2018) suggested an Event Leverage Framework to help community sport clubs leverage sport events taking place in their community, and Misener (2015) has developed the Parasport Leveraging Framework, which is a framework for leveraging parasport events for sport participation. None of the articles included in this review use these models to inform the design of the leveraging initiative. This means that although frameworks exist to guide leveraging initiatives for sport participation from sport events, they are not being utilized. Moreover, the lack of application makes it difficult to assess the effectiveness of these proposed frameworks. This lack of application of research-informed frameworks could be a contributing factor to the lack of research evidence to support the ability of sport events to stimulate sport participation. As such, stronger efforts to increase the ease of use of such frameworks by researchers are needed.

#### Study Design

**Figure 4:**
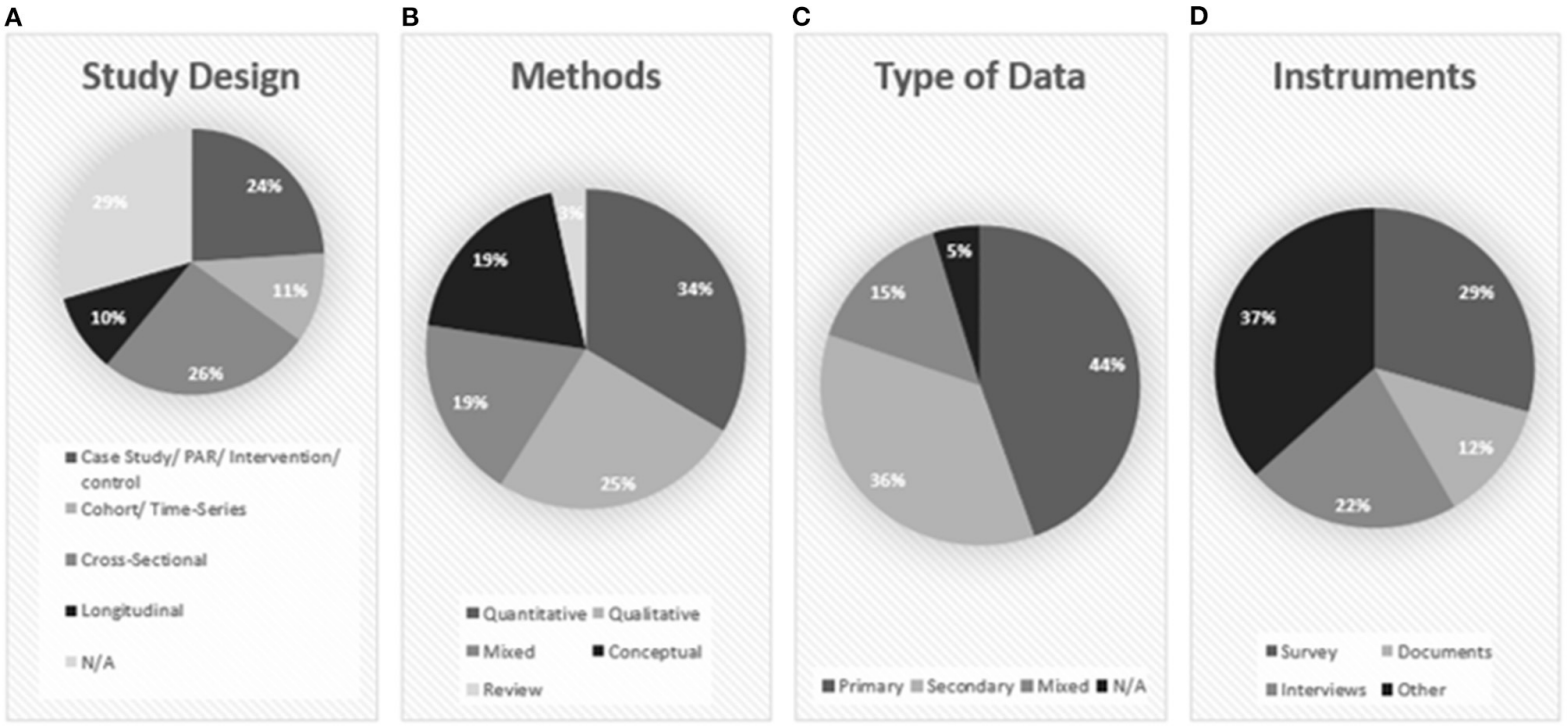
Research design: Study design **(A)**, Methods, **(B)**, Type of data **(C)**, and Instruments **(D)**.

A large portion (add 26%) of the studies included in this analysis have been cross-sectional in nature (Figure 4A). Very few have been longitudinal, making it difficult for researchers to make causal claims about the impacts of sport events. Even when studies are longitudinal, they tend to only use two time points, typically a pre-event and a post-event measure (e.g., Lane et al., 2012; Ramchandani et al., 2017b). Though the pre–post event design allows researchers to collect baseline data to highlight potential post-event changes in sport participation, multiple pre-event snapshots will allow for a clearer understanding of baseline, and multiple post-event assessments will allow for the long-term effects of potential impacts to be understood. The use of multiple time points (i.e., three or more) is necessary to understand the role that the specific event plays in any sport participation changes (Creswell, 2014). This type of longitudinal data would allow researchers to track possible behavioral changes as well as the nature of participation among the study population while considering important contextual factors such as the presence of a sport event.

Moreover, researchers can also consider the lead-up to events in stimulating sport participation. Some studies have found that sport participation increases leading up to the Olympic Games but is not sustained (e.g., Wang and Theodoraki, 2007; Bauman et al., 2013), supporting the notion of Weed et al. (2015) on the pregnancy effect. Future research may want to consider the specific aspects of the pre-event atmosphere that stimulate sport participation and how to prolong those conditions to sustain participation.

#### Methods

The largest portion of studies employs a quantitative approach (*n* = 49; 34%), including the use of nationally collected data (Figure 4B). Interestingly, Britain's Active Peoples Survey is used in a number of studies, coinciding with the theme of the London 2012 Olympics being heavily investigated (e.g., Bullough, 2012; Hughes, 2012; Carmichael et al., 2013). Although there has been a mix of qualitative (*n* = 37; 25%), mixed methods (*n* = 27; 19%), and conceptual (*n* = 33; 22%) approaches to examining sport participation impacts of sport events, the use of more mixed methods research to investigate impacts of events might be helpful, as different data sources will provide an alternative perspective on a phenomenon (Creswell, 2014). For instance, a longitudinal, mixed methods design could be executed in the area or region where the event takes place. Community members (particularly youth), sport providers, and policymakers could all be interviewed pre- and post-event to better understand how the event (may/has) affected sport participation (or not) and why. Simultaneously, a longitudinal, quantitative approach can be executed in the same region, collecting benchmark measures pre-event as well as data about event experiences and post-event effect measures.

Moreover, it has been well-established in the literature that sport events are not likely to lead to mass-participation changes (e.g., McCartney et al., 2010; Weed et al., 2015; Annear et al., 2019), but rather targeted leveraging initiatives within specific contexts and targeted populations are needed (e.g., Misener, 2015; Chalip et al., 2017; Taks et al., 2018). As such, we encourage researchers to move away from population-level data and take on research methods that can support research aims that are more targeted to better understand and establish leveraging mechanisms in host communities. Such approaches can include participatory action research where researchers deliberately assist (sport) organizations with developing, implementing, and evaluating leveraging initiatives tied to events in their community.

#### Type of Data

The use of primary (*n* = 65; 44%) and secondary (*n* = 52; 36%) data is fairly evenly distributed among the studies included in this scoping review, along with studies that employ both types of data (*n* = 22; 15%) (Figure 4C). There is a trend of the studies published in the early 2010s that investigated past events to see if there was a population-level change associated with major events. These studies, of course, would have used secondary data, which explains why there are so many studies in that category. For instance, Bauman et al. (2015) used population-level data to examine if there had been an increase in sport and physical activity among Australians immediately following the Sydney 2000 Olympic Games. Similar studies were conducted with regard to the Greek population and the Athens 2004 Olympics (Pappous, 2011) and Canadians and the Vancouver 2010 Olympic Games (Perks, 2015).

The specific data collection instruments employed across all empirical studies (*n* = 104) include surveys, with 43 studies employing primary data collection surveys and 10 using secondary survey data. Interviews (*n* = 29) and document analyses (*n* = 22) are also a popular means of data collection (Figure 4D). Less popular methods of data collection that have been employed are focus groups (*n* = 2), membership data (*n* = 4), observations (*n* = 4), and diaries (*n* = 4). The remaining studies were conceptual pieces (*n* = 42) and thus did not employ specific methods of data collection. In line with our previous suggestion to execute longitudinal, mixed methods approaches for these types of studies, combining various types of data simultaneously is strongly encouraged to address more targeted research aims. For example, questionnaires measuring attitudes toward sport and physical activity at multiple time points can be conducted with participants simultaneously with journal entries to elaborate on cognitive and affective states while engaging with the sport event. Moreover, to assist youth in expressing their thoughts (Taylor, 2013), art-based methods of data collection can also be considered.

### Research Findings

**Figure 5:**
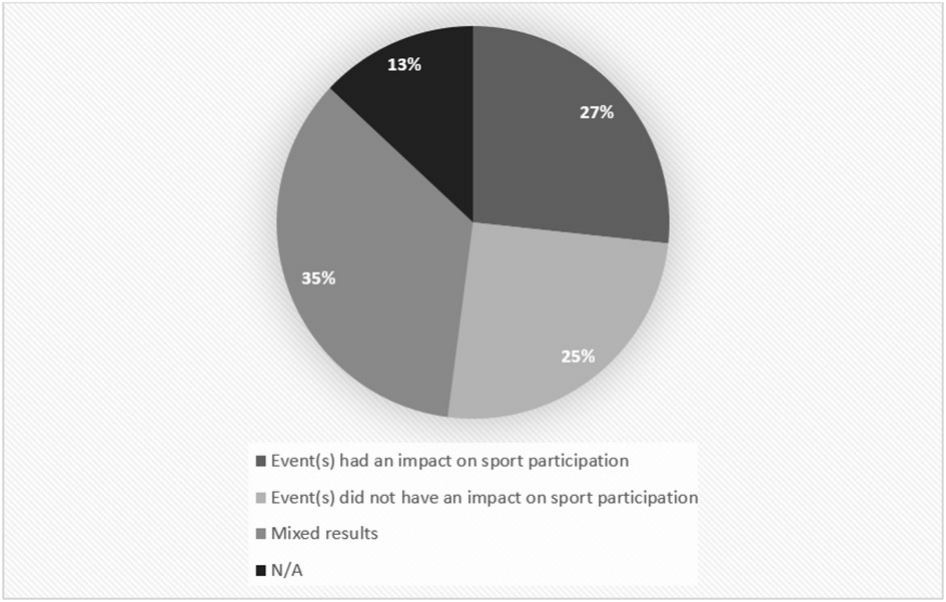
Research findings.

About a quarter of the studies supported that the event under investigation could positively impact sport participation (*n* = 39; 27%); another quarter found that the sport event under investigation did not impact sport participation (*n* = 37; 25%); and 51 (35%) of the studies found mixed results, meaning that the event in the study under investigation had an impact on participation under specific conditions and not in others, or it was not clear if the event had an impact. The remaining 13% (*n* = 19) were commentary articles and thus did not include findings (Figure 5). Note that impact, outcomes, legacies, and leveraging were not used as an extraction category. All papers with research findings related to any of these four concepts were included in this review. What is noticeable is that delineation between impacts, outcomes, and legacies were not always clear; these concepts were often used interchangeably. In studies reporting on leveraging, the concept was generally clearly defined, but these articles were limited and in an early stage of conceptual and theoretical development. Indeed, for the line of research addressing sport participation from sport events, clear definitions of the nature of sport participation impacts, outcomes, and leveraging should be established.

#### Youth vs. Adult Populations

In terms of both adult and youth populations, the results for sport participation outcomes are mixed. For instance, there was no increase in sport participation among Australian adults following the Sydney 2000 Olympic Games (Bauman et al., 2015), but Aizawa et al. (2018) found that the cohort of Japanese citizens that experienced the 1964 Tokyo Olympics were more active than those who had not. However, the causality of this relationship is unclear, since other initiatives (e.g., policies) may have stimulated participation for that particular generation.

For a youth population, Canadian female youth living in regions that housed Olympic venues where Canadian female athletes performed well during the 2010 Vancouver Olympics experienced significant increases in sport participation post-event, whereas male youth in the same regions and both male and female youth outside of the regions did not experience a significant change in participation (Potwarka and Leatherdale, 2016). It should be noted, however, that increased participation in this case does not necessarily mean that nonparticipants take up sport (i.e., current participants could be participating at higher rates or trying different sports).

#### Spectator vs. Participant Events

In terms of live spectatorship of elite sports, both Ramchandani and Coleman (2012) and Potwarka et al. (2017) found that the live event inspired spectators to increase their sport participation. Potwarka et al. (2017) found that cognitive dimensions of the spectator experience (i.e., fantasy, flow, evaluation, esthetics, and physical attractiveness) led to the inspiration, though inspiration does not guarantee participation. Ramchandani and Coleman (2012) suggested that spectators must be provided with information on how to change or increase participation based on inspiration from attending sport events. Indeed, much of the support for spectator events has been found in the live-spectatorship context. There remains much to be investigated about how spectator events can impact sport participation in terms of whether these impacts exist, to what extent, and under what circumstances.

Although there are less studies (*N* = 26; 18%) investigating participant events (Figure 3), participant events seem to be more effective at increasing sport participation than their spectator counterparts; the majority of studies (*n* = 18) concluded that the event did indeed have a positive impact on sport participation. In terms of participant events, Stevinson and Hickson (2014) found that a fun run was effective in attracting non-runners to participate. Moreover, Bowles et al. (2006) found that for the novice participants and first-time participants of a cycling event, there was a significant increase in post-event participation. Finally, Crofts et al. (2012) found that shorter distance participant events have the potential to lead to future participation in other participant events. Seemingly, participant events have a stronger potential than spectator sport events to stimulate new participation in sport. Events can impact sport participation before, during, and after the event takes place, and consistent with previous research on participant events (e.g., Funk et al., 2011), participant events seem to be effective in stimulating sport participation in that participants train in preparation for the event and intend to remain active post-event (e.g., Bowles et al., 2006; Crofts et al., 2012; Stevinson and Hickson, 2014; Coleman and Sebire, 2017) possibly in preparation for the next participant event (Crofts et al., 2012).

#### Mega/Large vs. Small/Medium-Sized Events

Generally speaking, there is little evidence to support that mega sport events stimulate grassroot participation. Toohey (2008) found that the policies and funding for sport around the 2000 Olympic Games in Australia heavily favored elite athletes at the expense of grassroots participation. With respect to the London 2012 Olympic Games, few organizations were willing to take on the responsibility of delivering a sport participation legacy (Bloyce and Lovett, 2012), and those eventually charged with delivering the legacy (e.g., community sport clubs) lack the resources to do so (Hughes, 2012). The lack of support for sport impacts from mega sport events has largely been conceptualized as an “unmet legacy” (Bloyce and Lovett, 2012; Hughes, 2012; Orr, 2018).

Grix et al. (2017) found that when London was awarded the bid to host the 2012 Olympic Games, and leading up to the Games, there was an increase in participation among the London population, though participation levels declined back to pre-bid levels. Interestingly, Carter and Lorenc (2015) found that watching the elite athletes during the London 2012 Olympic Games could actually discourage inactive adults from participating in sport, while watching mass participation events and Paralympic athletes could serve to inspire participation.

Small and medium spectator events show mixed results as to whether or not the event had stimulated sport participation, as two studies concluded that the event under investigation had positively impacted sport participation (i.e., Ramchandani and Coleman, 2012; Ramchandani et al., 2015), while three studies concluded that the event had no impact on sport participation (i.e., Taks et al., 2014; Hodgetts and Duncan, 2015; Misener et al., 2015b). Interestingly, six of the studies found mixed results in that there was not enough evidence to support a claim of increased participation or there was increased participation among certain populations and no changes in participation among others (i.e., Taks et al., 2013, 2015, 2018; Derom and VanWynsberghe, 2015; Misener, 2015; Hoskyn et al., 2018). There is an additional gap with regard to the role of smaller sport events in contributing to sport participation outcomes. As suggested by Taks (2013), smaller events may be more powerful in improving social benefits to host communities, including sport participation. Thus, an additional line of research should address the role of small events in impacting sport participation.

#### Effect of Event Leveraging

Effects of event leveraging studies looking into how to effectively leverage non-mega sport events for sport participation impacts show mixed results (Misener et al., 2015a; Chalip et al., 2017; Hoskyn et al., 2018; Taks et al., 2018). Effective leveraging strategies have yet to be identified, with results largely indicating that sport clubs lack the capacity to engage in leveraging strategies (Taks et al., 2018) or event attendees engage minimally with leveraging initiatives (Hoskyn et al., 2018).

Future research should attempt to establish best practices for event leveraging and identify benchmarks for event practitioners to draw from. Furthermore, drawing from marketing theory (Kotler and Keller, 2009), the use of similar strategies to elicit participation at a population level is ineffective; different populations need different stimuli to elicit participation outcomes.

### Limitations

As scoping reviews do not require an appraisal of research quality (Arksey and O'Malley, 2005), some of the articles included in the present scoping review could be of lower quality. Moreover, scoping reviews also do not involve a synthesis of research findings (Arksey and O'Malley, 2005), thus this project only briefly highlighted which types and sizes of sport events generate which outcomes, without addressing the ways that sport events can affect sport participation (if at all).

## Conclusion

A scoping review was conducted to assess the current state of literature that addresses impacts, legacies, and leveraging of sport events for sport participation outcomes and the gaps in terms of study context and research designs. A summary of key findings discussed can be found in Table 3.

**Table 3:** Summary of key findings.

**Trend**	**Gap**	**Direction**
Emphasis on mega and large events	Small and medium events are unresearched	Consider small and medium events when investigating sport participation from sport events
Major multisport events studied without the inclusion of their parasport counterparts and *vice versa*	Perhaps missing overall impact of event	Consider investigating both event contexts
Emphasis on spectator events	Still much to be researched with regard to the power of participant events to impact sport participation	Consider participant events or incorporating participant events alongside spectator events
Emphasis on adult sport participation and sport organization's perspectives	Little research on youth populations and marginalized populations	Targeted research agenda for youth and marginalized populations
Inconsistent use of theory	Lack of theory-driven research	Adopt theoretical approaches when investigating sport participation from sport events
Emphasis on cross-sectional research	Lack of causal insights	Employ longitudinal, mixed methods approaches
Population-level data are often used	More targeted research methods can be used	Take on research methods that can support research aims that are more targeted
Mixed results about the ability of sport events to impact sport participation	Nature of sport participation from sport events remains unclear	Continue research into effective leveraging strategies

Studies looking into the potential impacts of sport events on sport participation tend to be cross-sectional in nature and primarily investigate adult populations or the population as a whole. These studies also tend to look into mega spectator-based events. This scoping review has provided several key insights for a research agenda for sport management scholars in the area of sport participation from sport events to subsequently inform sport policy and event planners and organizations. Moving forward, researchers should employ longitudinal investigations that collect data at multiple time points to help understand the specific role that the event plays in shaping attitudes, intentions, and behaviors toward sport participation. These investigations should also be more theory-driven. Specifically, behavior change theories may enhance our understanding of it and how behavioral outcomes (e.g., attitudes, intentions, and behaviors) toward sport participation are generated from sport events. However, given the use of limited, but various, theoretical lenses, a grounded-theory approach to exploring the phenomenon could also be considered to develop a theory of sport participation from sport events.

Furthermore, as many claims made about the impacts of sport events are made with regard to youth populations, future investigations should be conducted with these youth populations to better understand the role of the event with regard to youth. Moreover, and particularly relevant to sport management practitioners, participant events should also be considered as effective ways of increasing sport participation through events. If an important goal of hosting sport events is to increase participation in sport, hosting participant events should be considered, rather than spectator events, as this scoping review indicates that participant events may have more potential than spectator events in this regard. Alternatively, traditional spectator events, such as Olympics or PanAmerican Games, can consider implementing participant-based aspects, such as a mass participant event to help open the Games where citizens of the host region are invited to participate (e.g., Derom et al., 2015). Finally, more research is needed with regard to the role of small and medium sport events, both participant and spectator, in sport participation outcomes. As we can see from this scoping review, smaller participant events seem to have potential to increase sport participation; however, they are also underresearched. In summary, we recommend further lines of inquiry into the role and mechanisms of smaller events, both spectator and participant events, in impacting sport participation, as well as a focus on youth. Taking a longitudinal approach is also highly recommended.

## Author Contributions

All authors listed have made a substantial, direct and intellectual contribution to the work, and approved it for publication.

## Conflict of Interest

The authors declare that the research was conducted in the absence of any commercial or financial relationships that could be construed as a potential conflict of interest.

## References

[B1] Aizawa K. Wu J. Inoue Y. Sato M. (2018). Long-term impact of the Tokyo 1964 Olympic Games on sport participation: a cohort analysis. Sport Manag Rev. 21, 86–97. 10.1016/j.smr.2017.05.001

[B2] Allender S. Cowburn G. Foster C. (2006). Understanding participation in sport and physical activity among children and adults: a review of qualitative studies. Health Educ. Res. 21, 826–835. 10.1093/her/cyl063 16857780

[B3] Annear M. J. Shimizu Y. Kidokoro T. (2019). Sports mega-event legacies and adult physical activity: a systematic literature review and research agenda. Eur. J. Sport Sci. 19, 671–685. 10.1080/17461391.2018.1554002 30556493

[B4] Arksey H. O'Malley L. (2005). Scoping studies: towards a methodological framework. Int. J. Soc. Res. Methodol. 8, 19–32. 10.1080/1364557032000119616

[B5] Bauman A. Bellew B. Craig C. L. (2015). Did the 2000 Sydney Olympics increase physical activity among adult Australians? Br. J. Sports Med. 49, 243–247. 10.1136/bjsports-2013-093149 24831816

[B6] Bauman A. E. Murphy N. Matsudo V. (2013). Is a population-level physical activity legacy of the London 2012 Olympics likely? J. Phys. Act. Health 10, 1–4. 10.1123/jpah.10.1.1 22398508

[B7] Bell B. Daniels J. (2018). Sport development in challenging times: leverage of sport events for legacy in disadvantaged communities. Manag. Sport Leis. 23, 369–390. 10.1080/23750472.2018.1563497

[B8] Black A. Costello R. Craft A. Katene W. (2015). ‘It's all about developing the whole child': an examination of the ‘legacy' benefits of Youth Sport Trust's school-based inclusion initiatives. Eur. Phys. Educ. Rev. 21, 362–378. 10.1177/1356336X15584089

[B9] Bloyce D. Lovett E. (2012). Planning for the London 2012 Olympic and Paralympic legacy: a figurational analysis. Int. J. Sport Policy Polit. 4, 361–377. 10.1080/19406940.2012.740063

[B10] Bowles H. R. Rissel C. Bauman A. (2006). Mass community cycling events: who participates and is their behaviour influenced by participation? Int. J. Behav. Nutr. Phys. Act. 3:39. 10.1186/1479-5868-3-39 17090328 PMC1647288

[B11] Brown C. Pappous A. (2018). “The Legacy Element. It Just Felt More Woolly”: exploring the reasons for the decline in people with disabilities' sport participation in England 5 years after the London 2012 Paralympic Games. J. Sport Soc. Issues 42, 343–368. 10.1177/0193723518781237

[B12] Brown G. Essex S. Assaker G. Smith A. (2017). Event satisfaction and behavioural intentions: examining the impact of the London 2012 Olympic Games on participation in sport. Eur. Sport Manag. Q. 17, 331–348. 10.1080/16184742.2017.1294193

[B13] Bullough S. J. (2012). A new look at the latent demand for sport and its potential to deliver a positive legacy for London 2012. Int. J. Sport Policy Polit. 4, 39–54. 10.1080/19406940.2011.627357

[B14] Carmichael F. Grix J. Marqués D. P. (2013). The Olympic legacy and participation in sport: an interim assessment of Sport England's Active People Survey for sports studies research. Int. J. Sport Policy Polit. 5, 229–244. 10.1080/19406940.2012.656675

[B15] Carter R. V. Lorenc T. (2015). A qualitative study into the development of a physical activity legacy from the London 2012 Olympic Games. Health Promot. Int. 30, 793–802. 10.1093/heapro/dat066 24052334

[B16] Chalip L. (2006). Towards social leverage of sport events. J. Sport Tour. 11, 109–127. 10.1080/14775080601155126

[B17] Chalip L. (2014). “From legacies to leverage,” in Leveraging Legacies From Sports Mega-Events: Concepts and Cases, ed Grix J (London: Palgrave Pivot), 2–12.

[B18] Chalip L. Green B. C. Taks M. Misener L. (2017). Creating sport participation from sport events: Making it happen. Int. J. Sport Policy Polit. 9, 257–276. 10.1080/19406940.2016.1257496

[B19] Charlton T. (2010). ‘Grow and Sustain': the role of community sports provision in promoting a participation legacy for the 2012 Olympic Games. Int. J. Sport Policy Polit. 2, 347–366. 10.1080/19406940.2010.519340

[B20] Chen S. Henry I. (2016). Evaluating the London 2012 Games' impact on sport participation in a non-hosting region: a practical application of realist evaluation. Leis. Stud. 35, 685–707. 10.1080/02614367.2015.1040827

[B21] Coalter F. (2004). “London 2012: a sustainable sporting legacy?,” in After the Goldrush: A Sustainable Olympics for London, eds Vigor A Mean M (London: ippra Demos), 14.

[B22] Coates J. Vickerman P. B. (2016). Paralympic legacy: exploring the impact of the Games on the perceptions of young people with disabilities. Adapt. Phys. Act Q. 33, 338–357. 10.1123/APAQ.2014-0237 27874305

[B23] Coleman S. J. Sebire S. J. (2017). Do people's goals for mass participation sporting events matter? A self-determination theory perspective. J. Public Health 39, E202–E208. 10.1093/pubmed/fdw090 27679656

[B24] Craig C. L. Bauman A. E. (2014). The impact of the Vancouver Winter Olympics on population level physical activity and sport participation among Canadian children and adolescents: population based study. Int. J. Behav. Nutr. Phys. Act. 11:107. 10.1186/s12966-014-0107-y 25182041 PMC4180145

[B25] Creswell J. W. (2014). Research Design: Qualitative, Quantitative, and Mixed Methods Approaches, 4th Edn. Thousan Oaks, CA: SAGE.

[B26] Crofts C. Schofield G. Dickson G. (2012). Women-only mass participation sporting events: does participation facilitate changes in physical activity? Ann. Leis. Res. 15, 148–159. 10.1080/11745398.2012.685297

[B27] Darko N. Mackintosh C. (2016). ‘Don't you feel bad watching the Olympics, watching us?' A qualitative analysis of London 2012 Olympics influence on family sports participation and physical activity. Qual. Res. Sport Exerc. Health 8, 45–60. 10.1080/2159676X.2015.1056825

[B28] Derom I. VanWynsberghe R. (2015). Extending the benefits of leveraging cycling events: evidence from the Tour of Flanders. Eur. Sport Manag Q. 15, 111–131. 10.1080/16184742.2014.997772

[B29] Derom I. VanWynsberghe R. Scheerder J. (2015). Maintaining physical activity post-event? Case of the Tour of Flanders Cyclo in Belgium. Ann. Leis. Res. 18, 25–47. 10.1080/11745398.2014.932699

[B30] Dowling M. Leopkey B. Smith L. (2018). Governance in sport: a scoping review. J. Sport Manag. 32, 438–451. 10.1123/jsm.2018-0032

[B31] Dubnewick M. Hopper T. Spence J. C. McHugh T.-L. F. (2018). “There's a Cultural Pride Through Our Games”: enhancing the sport experiences of Indigenous youth in Canada through participation in Traditional Games. J. Sport Soc Issues 42, 207–226. 10.1177/0193723518758456

[B32] Filo K. Lock D. Karg A. (2015). Sport and social media research: a review. Sport Manag Rev. 18, 166–181. 10.1016/j.smr.2014.11.001

[B33] Funk D. Jordan J. Ridinger L. Kaplanidou K. (2011). Capacity of mass participant sport events for the development of activity commitment and future exercise intention. Leis. Sci. 33, 250–268. 10.1080/01490400.2011.564926

[B34] Gammon S. (2012). “Sports events: typologies, people and place,” in The Routledge Handbook of Events, eds Page SJ Connell J (London: Routledge), 104–118.

[B35] Geurin A. N. Naraine M. L. (2020). 20 years of Olympic media research: trends and future directions. Front. Sports Act Liv. 2:572495. 10.3389/fspor.2020.572495 33345133 PMC7739588

[B36] Girginov V. (2016). Has the London 2012 Olympic Inspire programme inspired a generation? A realist view. Eur. Phys. Educ. Rev. 22, 490–505. 10.1177/1356336X15623169

[B37] Grant M. J. Booth A. (2009). A typology of reviews: an analysis of 14 review types and associated methodologies. Health Inf. Libr. J. 26, 91–108. 10.1111/j.1471-1842.2009.00848.x 19490148

[B38] Griffiths M. Armour K. (2013). Physical education and youth sport in England: conceptual and practical foundations for an Olympic legacy? Int. J. Sport Policy Polit. 5, 213–227. 10.1080/19406940.2012.656676

[B39] Grix J. Brannagan P. M. Wood H. Wynne C. (2017). State strategies for leveraging sports mega-events: Unpacking the concept of ‘legacy.' Int. J. Sport Policy Polit. 9, 203–218. 10.1080/19406940.2017.1316761

[B40] Hayday E. J. Pappous A. Koutrou N. (2017). Leveraging the sport participation legacy of the London 2012 Olympics: senior managers' perceptions. Int. J. Sport Policy Polit. 9, 349–369. 10.1080/19406940.2016.1255241

[B41] Hindson A. Gidlow B. Peebles C. (1994). The ‘trickle-down'effect of top level sport: myth or reality? A case study of the Olympics. Aust. J. Leis. Recreat. 4, 16–24.

[B42] Hodgetts D. Duncan M. J. (2015). Quantitative analysis of sport development event legacy: an examination of the Australian Surf Life Saving Championships. Eur. Sport Manag Q. 15, 364–380. 10.1080/16184742.2015.1021824

[B43] Hoskyn K. Dickson G. Sotiriadou P. (2018). Leveraging medium-sized sport events to attract club participants. Mark. Intell. Plan. 36, 199–212. 10.1108/MIP-04-2017-0063

[B44] Hughes K. (2012). “Mega sports events and the potential to create a legacy of increased sport participation in the host country,” in International Sports Events: Impacts, Experiences and Identities, eds Shipway R. Fyall A. (New York, NY: Routledge), 42–54.

[B45] Inoue Y. Berg B. K. Chelladurai P. (2015). Spectator sport and population health: a scoping study. J. Sport Manag. 29, 705–725. 10.1123/JSM.2014-0283

[B46] Kohe G. Z. (2017). London 2012 (Re)calling: youth memories and Olympic ‘legacy' ether in the hinterland. Int. Rev. Sociol. Sport. 52, 24–44. 10.1177/1012690215581604

[B47] Kohe G. Z. Bowen-Jones W. (2016). Rhetoric and realities of London 2012 Olympic education and participation ‘legacies': Voices from the core and periphery. Sport Educ. Soc. 21, 1213–1229. 10.1080/13573322.2014.997693

[B48] Kotler P. Keller K. L. (2009). Marketing Management, 13th Edn. Pearson.

[B49] Lane A. Murphy N. Bauman A. Chey T. (2012). Active for a day: predictors of relapse among previously active mass event participants. J. Phys. Act. Health 9, 48–52. 10.1123/jpah.9.1.48 22232504

[B50] Lovett E. Bloyce D. (2017). What happened to the legacy from London 2012? A sociological analysis of the processes involved in preparing for a grassroots sporting legacy from London 2012 outside of the host city. Sport Soc. 20, 1625–1643. 10.1080/17430437.2017.1284813

[B51] Mackintosh C. Darko N. Rutherford Z. Wilkins H.-M. (2015). A qualitative study of the impact of the London 2012 Olympics on families in the East Midlands of England: lessons for sports development policy and practice. Sport Educ. Soc. 20, 1065–1087. 10.1080/13573322.2014.881337

[B52] McCartney G. Thomas S. Thomson H. Scott J. Hamilton V. Hanlon P.. (2010). The health and socioeconomic impacts of major multi-sport events: systematic review (1978-2008). BMJ 340:c2369. 10.1136/bmj.c2369 20488915 PMC2874130

[B53] Misener L. (2015). Leveraging parasport events for community participation: development of a theoretical framework. Eur. Sport Manag Q. 15, 132–153. 10.1080/16184742.2014.997773

[B54] Misener L. McGillivray D. McPherson G. Legg D. (2015a). Leveraging parasport events for sustainable community participation: The Glasgow 2014 Commonwealth Games. Ann. Leis. Res. 18, 450–469. 10.1080/11745398.2015.1045913

[B55] Misener L. Taks M. Chalip L. Green B. C. (2015b). The elusive “trickle-down effect” of sport events: assumptions and missed opportunities. Manag. Sport Leis. 20, 135–156. 10.1080/23750472.2015.1010278

[B56] Müller M. (2015). The mega-event syndrome: why so much goes wrong in mega-event planning and what to do about it. J. Am. Plann. Assoc. 81, 6–17. 10.1080/01944363.2015.1038292

[B57] Orr M. (2018). Blinded by gold: Toronto sports community ignores negative legacies of 2015 Pan Am Games. Event Manag. 22, 367–378. 10.3727/152599518X15252008713173

[B58] Pappous A. (2011). “Do the olympic games lead to a sustainable increase in grassroots sport participation? A secondary analysis of Athens 2004,” in Sustainability and Sport, eds Savery J. Gilbert K.(Champaign, IL: Common Ground), 81–87.

[B59] Perks T. (2015). Exploring an Olympic “legacy”: sport participation in Canada before and after the 2010 Vancouver Winter Olympics. Can. Rev. Sociol. Can. Sociol. 52, 462–474. 10.1111/cars.12087 26577884

[B60] Potwarka L. R. Drewery D. Snelgrove R. Havitz M. E. Mair H. (2017). Modeling a demonstration effect: the case of spectators' experiences at 2015 Pan Am Games' track cycling competitions. Leis. Sci. 40, 578–600. 10.1080/01490400.2017.1325796

[B61] Potwarka L. R. Leatherdale S. T. (2016). The Vancouver 2010 Olympics and leisure-time physical activity rates among youth in Canada: Any evidence of a trickle-down effect? Leis. Stud. 35, 241–257. 10.1080/02614367.2015.1040826

[B62] Potwarka L. R. Wicker P. (2021). Conditions under which Trickle-Down Effects occur: a Realist Synthesis approach. Sustainability 13:69. 10.3390/su13010069

[B63] Preuss H. (2019). Event legacy framework and measurement. Int. J. Sport Policy Polit. 11, 103–118. 10.1080/19406940.2018.1490336

[B64] Ramchandani G. Coleman R. Christy E. (2017a). The sport participation legacy of major events in the UK. Health Promot Int. 34, 82–94. 10.1093/heapro/dax061 28973157

[B65] Ramchandani G. Coleman R. J. Bingham J. (2017b). Sport participation behaviours of spectators attending major sports events and event induced attitudinal changes towards sport. Int. J. Event Festiv. Manag Bingley 8, 121–135. 10.1108/IJEFM-02-2016-0014

[B66] Ramchandani G. Davies L. E. Coleman R. Shibli S. Bingham J. (2015). Limited or lasting legacy? The effect of non-mega sport event attendance on participation. Eur. Sport Manag. Q. 15, 93–110. 10.1080/16184742.2014.996583

[B67] Ramchandani G. M. Coleman R. J. (2012). The inspirational effects of three major sport events. Int. J. Event Festiv. Manag. Bingley 3, 257–271. 10.1108/17582951211262693

[B68] Sandercock G. R. H. Beedie C. Mann S. (2016). Is Olympic inspiration associated with fitness and physical activity in English schoolchildren? A repeated cross-sectional comparison before and 18 months after London 2012. BMJ Open 6:e011670. 10.1136/bmjopen-2016-011670 27881520 PMC5168498

[B69] Selvanayagam M. Thompson C. Taylor S. J. C. Cummins S. Bourke L. (2012). How might the London 2012 Olympics influence health and the determinants of health? Local newspaper analysis of pre-Games pathways and impacts. BMJ Open 2:e001791. 10.1136/bmjopen-2012-001791 23151394 PMC3533038

[B70] Silvey D. Buote R. Donovan C. Dubrowski A. (2018). Impacts of the 2018 Newfoundland and Labrador Winter Games on youth who participated in the sport of Olympic Wrestling with Team Indigenous. Phys. Health Educ. J. Glos. 84, 1–20.

[B71] Smith N. R. Lewis D. J. Fahy A. Eldridge S. Taylor S. J. Moore D. G.. (2015). Individual socio-demographic factors and perceptions of the environment as determinants of inequalities in adolescent physical and psychological health: the Olympic Regeneration in East London (ORiEL) study. BMC Public Health 15:150. 10.1186/s12889-015-1459-1 25884502 PMC4339478

[B72] Stevinson C. Hickson M. (2014). Exploring the public health potential of a mass community participation event. J. Public Health. 36, 268–274. 10.1093/pubmed/fdt082 23954885

[B73] Stokols D. (1992). Establishing and maintaining healthy environments: toward a social ecology of health promotion. Am. Psychol. 47, 6–22. 10.1037/0003-066X.47.1.6 1539925

[B74] Such E. (2016). The Olympic family? Young people, family practices and the London 2012 Olympic Games. Int. J. Sport Policy Polit. 8, 189–206. 10.1080/19406940.2015.1105278

[B75] Taks M. (2013). Social sustainability of non-mega sport events in a global world. Eur. J. Sport Soc. 10, 121–141. 10.1080/16138171.2013.11687915

[B76] Taks M. Chalip L. Green B. C. (2015). Impacts and strategic outcomes from non-mega sport events for local communities. Eur. Sport Manag. Q. 15, 1–6. 10.1080/16184742.2014.995116

[B77] Taks M. Green B. C. Misener L. Chalip L. (2014). Evaluating sport development outcomes: the case of a medium-sized international sport event. Eur. Sport Manag. Q. 14, 213–237. 10.1080/16184742.2014.882370

[B78] Taks M. Green B. C. Misener L. Chalip L. (2018). Sport participation from sport events: why it doesn't happen? Mark. Intell. Plan. 36, 185–198. 10.1108/MIP-05-2017-0091

[B79] Taks M. Misener L. Chalip L. Green B. C. (2013). Leveraging sport events for participation. Can. J. Soc. Res. 3, 12–23.

[B80] Taylor B. (2013). “Creative form of qualitative data collection and analysis,” in Qualitative Research in the Health Sciences: Methodologies, Methods and Processes, 1st Edn, eds Taylor B. Francis K. (Abingdon, VA: Routledge), 266–284.

[B81] Teare G. Taks M. (2020). Extending the scoping review framework: a guide for interdisciplinary researchers. Int. J. Soc. Res. Methodol. 23, 311–315. 10.1080/13645579.2019.1696092

[B82] Thompson A. Cuskelly G. Toohey K. Kennelly M. Burton P. Fredline L. (2019). Sport event legacy: a systematic quantitative review of literature. Sport Manag. Rev. 22, 295–321. 10.1016/j.smr.2018.06.011

[B83] Thomson A. Kennelly M. Toohey K. (2020). A systematic quantitative literature review of empirical research on large-scale sport events' social legacies. Leis. Stud. 39, 859–876. 10.1080/02614367.2020.1800804

[B84] Toohey K. (2008). The Sydney Olympics: striving for legacies – overcoming short-term disappointments and long-term deficiencies. Int. J. Hist. Sport 25, 1953–1971. 10.1080/09523360802439270

[B85] Veal A. J. Toohey K. Frawley S. (2012). The sport participation legacy of the Sydney 2000 Olympic Games and other international sporting events hosted in Australia. J. Policy Res. Tour Leis. Events 4, 155–184. 10.1080/19407963.2012.662619

[B86] Wang W. Theodoraki E. (2007). Mass sport policy development in the Olympic City: the case of Qingdao — host to the 2008 sailing regatta. J. R. Soc. Promot. Health 127, 125–132. 10.1177/1466424007077345 17542425

[B87] Weed M. Coren E. Fiore J. Wellard I. Chatziefstathiou D. Mansfield L.. (2015). The Olympic Games and raising sport participation: a systematic review of evidence and an interrogation of policy for a demonstration effect. Eur. Sport Manag. Q. 15, 195–226. 10.1080/16184742.2014.998695

[B88] Wellings K. Datta J. Wilkinson P. Petticrew M. (2011). The 2012 Olympics: assessing the public health effect. Lancet 378, 1193–1195. 10.1016/S0140-6736(11)60550-3 21798591

[B89] Whittemore R. Chao A. Jang M. Minges K. E. Park C. (2014). Methods for knowledge synthesis: an overview. Heart Lung 43, 453–461. 10.1016/j.hrtlng.2014.05.014 25012634

