# Supplementary material for: Sport Events for Sport Participation: A Scoping Review
Source: Front Sports Act Living. 2021 May 19;3:655579. doi: 10.3389/fspor.2021.655579 (PMC8170037; doi:10.3389/fspor.2021.655579)
Supplement: Supplementary file 1 [file Table_1.DOCX]

Supplementary Material

# Supplementary Table

| Search Method | Citation |
| --- | --- |
| Both | Barrick, S., Mair, H. L., & Potwarka, L. R. (2017). Leveraging participation in Olympic sports: A call for experiential qualitative case study research. *Sport in Society*, *20*(12), 1861–1869. |
| Both | Bauman, Bellew, B., & Craig, C. L. (2015). Did the 2000 Sydney Olympics increase physical activity among adult Australians? *British Journal of Sports Medicine*, *49*(4), 243–247. |
| Both | Bauman, A. E., Murphy, N., & Matsudo, V. (2013). Is a population-level physical activity legacy of the London 2012 Olympics likely? *Journal of Physical Activity & Health*, *10*(1), 1–4. |
| Both | Boardley, I. D. (2013). Can viewing London 2012 influence sport participation? – A viewpoint based on relevant theory. *International Journal of Sport Policy and Politics*, *5*(2), 245–256. |
| Both | Bretherton, P., Piggin, J., & Bodet, G. (2016). Olympic sport and physical activity promotion: The rise and fall of the London 2012 pre-event mass participation ‘legacy.’ *International Journal of Sport Policy and Politics*, *8*(4), 609–624. |
| Both | Brown, G., Essex, S., Assaker, G., & Smith, A. (2017). Event satisfaction and behavioural intentions: Examining the impact of the London 2012 Olympic Games on participation in sport. *European Sport Management Quarterly*, *17*(3), 331–348. |
| Both | Brown, C., & Pappous, A. (2018). “The legacy element. . . It just felt more woolly”: Exploring the reasons for the decline in people with disabilities’ sport participation in England 5 years after the London 2012 Paralympic Games. *Journal of Sport and Social Issues*, *42*(5), 343–368. |
| Both | Bullough, S. J. (2012). A new look at the latent demand for sport and its potential to deliver a positive legacy for London 2012. *International Journal of Sport Policy and Politics*, *4*(1), 39–54. |
| Both | Carmichael, F., Grix, J., & Marqués, D. P. (2013). The Olympic legacy and participation in sport: An interim assessment of Sport England’s Active People Survey for sports studies research. *International Journal of Sport Policy and Politics*, *5*(2), 229–244. |
| Both | Carter, R. V., & Lorenc, T. (2013). A qualitative study into the development of a physical activity legacy from the London 2012 Olympic Games. *Health Promotion International*, *30*(3), 793–802. |
| Both | Chalip, L., Green, B. C., Taks, M., & Misener, L. (2017). Creating sport participation from sport events: Making it happen. *International Journal of Sport Policy and Politics*, *9*(2), 257–276. |
| Both | Charlton, T. (2010). ‘Grow and Sustain’: The role of community sports provision in promoting a participation legacy for the 2012 Olympic Games. *International Journal of Sport Policy and Politics*, *2*(3), 347–366. |
| Both | Chen, S., & Henry, I. (2016). Evaluating the London 2012 Games’ impact on sport participation in a non-hosting region: A practical application of realist evaluation. *Leisure Studies*, *35*(5), 685–707. |
| Both | Craig, C. L., & Bauman, A. E. (2014). The impact of the Vancouver Winter Olympics on population level physical activity and sport participation among Canadian children and adolescents: Population based study. *International Journal of Behavioral Nutrition and Physical Activity*, *11*(1). |
| Both | Crofts, C., Schofield, G., & Dickson, G. (2012). Women-only mass participation sporting events: Does participation facilitate changes in physical activity? *Annals of Leisure Research*, *15*(2), 148–159 |
| Both | Derom, I., & Lee, D. (2014). Vancouver and the 2010 Olympic Games: Physical activity for all? *Journal of Physical Activity & Health*, *11*(8), 1556–1564. |
| Both | Derom, I., & VanWynsberghe, R. (2015). Extending the benefits of leveraging cycling events: Evidence from the Tour of Flanders. *European Sport Management Quarterly*, *15*(1), 111–131. |
| Both | Derom, I., VanWynsberghe, R., & Scheerder, J. (2015). Maintaining physical activity post-event? Case of the Tour of Flanders Cyclo in Belgium. *Annals of Leisure Research*, *18*(1), 25–47. |
| Both | Devine, C. (2013). London 2012 Olympic legacy: A big sporting society? *International Journal of Sport Policy and Politics*, *5*(2), 257–279. |
| Both | Du, J., Jordan, J. S., & Funk, D. C. (2015). Managing mass sport participation: Adding a personal performance perspective to remodel antecedents and consequences of participant sport event satisfaction. *Journal of Sport Management*, *29*(6), 688–704. |
| Both | Feng, J., & Hong, F. (2013). The legacy: Did the Beijing Olympic Games have a long-Term impact on grassroots sport participation in Chinese Townships? *The International Journal of the History of Sport*, *30*(4), 407–421. |
| Both | Frawley, S., & Cush, A. (2011). Major sport events and participation legacy: The case of the 2003 Rugby World Cup. *Managing Leisure*, *16*(1), 65–76. |
| Both | Funk, D., Jordan, J., Ridinger, L., & Kaplanidou, K. (2011). Capacity of mass participant sport events for the development of activity commitment and future exercise intention. *Leisure Sciences*, *33*(3), 250–268. |
| Both | Girginov, V., & Hills, L. (2008). A sustainable sports legacy: Creating a link between the London Olympics and sports participation. *The International Journal of the History of Sport*, *25*(14), 2091–2116 |
| Both | Green, K. (2012). London 2012 and sports participation: The myths of legacy. *Significance*, *9*(3), 13–16. |
| Both | Griffiths, M., & Armour, K. (2013). Physical education and youth sport in England: Conceptual and practical foundations for an Olympic legacy? *International Journal of Sport Policy and Politics*, *5*(2), 213–227. |
| Both | Harris, S., & Houlihan, B. (2016). Implementing the community sport legacy: The limits of partnerships, contracts and performance management. *European Sport Management Quarterly*, *16*(4), 433–458. |
| Both | Hayday, E. J., Pappous, A., & Koutrou, N. (2017). Leveraging the sport participation legacy of the London 2012 Olympics: Senior managers’ perceptions. *International Journal of Sport Policy and Politics*, *9*(2), 349–369. |
| Both | Hodgetts, D., & Duncan, M. J. (2015). Quantitative analysis of sport development event legacy: An examination of the Australian Surf Life Saving Championships. *European Sport Management Quarterly*, *15*(3), 364–380. |
| Both | Kidd, B. (2013). The global sporting legacy of the Olympic Movement. *Sport in Society*, *16*(4), 491–502. |
| Both | Kohe, G. Z. (2017). London 2012 (Re)calling: Youth memories and Olympic ‘legacy’ ether in the hinterland. *International Review for the Sociology of Sport*, *52*(1), 24–44. |
| Both | Kohe, G. Z., & Bowen-Jones, W. (2016). Rhetoric and realities of London 2012 Olympic education and participation ‘legacies’: Voices from the core and periphery. *Sport, Education and Society*, *21*(8), 1213–1229. |
| Both | Li, Y., & Luk, Y. M. (2011). Impacts of the 4th East Asian games on residents’ participation in leisure sports and physical activities – the case of Macau, China. *Acta Geographica Slovenica*, *51*(2), 377–390. |
| Both | Lane, A., Murphy, N., Bauman, A., & Chey, T. (2010). Randomized controlled trial to increase physical activity among insufficiently active women following their participation in a mass event. *Health Education Journal*, *69*(3), 287–296. |
| Both | Lines, G. (2007). The impact of media sport events on the active participation of young people and some implications for PE pedagogy. *Sport, Education and Society*, *12*(4), 349–366. |
| Both | Lovett, E., & Bloyce, D. (2017). What happened to the legacy from London 2012? A sociological analysis of the processes involved in preparing for a grassroots sporting legacy from London 2012 outside of the host city. *Sport in Society*, *20*(11), 1625–1643 |
| Both | Mackintosh, C., Darko, N., Rutherford, Z., & Wilkins, H.-M. (2015). A qualitative study of the impact of the London 2012 Olympics on families in the East Midlands of England: Lessons for sports development policy and practice. *Sport, Education and Society*, *20*(8), 1065–1087. |
| Both | Macrae, E. H. R. (2017). Delivering sports participation legacies at the grassroots level: The voluntary sports clubs of Glasgow 2014. *Journal of Sport Management*, *31*(1), 15–26. |
| Both | Mahtani, K. R., Protheroe, J., Slight, S. P., Demarzo, M. M. P., Blakeman, T., Barton, C. A., … Roberts, N. (2013). Can the London 2012 Olympics ‘inspire a generation’ to do more physical or sporting activities? An overview of systematic reviews. *BMJ Open*, *3*(1), e002058. |
| Both | McCartney, G., Hanlon, P., & Bond, L. (2013). How will the 2014 Commonwealth Games impact on Glasgow’s health, and how will we know? *Evaluation*, *19*(1), 24–39. |
| Both | Minnaert, L. (2012). An Olympic legacy for all? The non-infrastructural outcomes of the Olympic Games for socially excluded groups (Atlanta 1996–Beijing 2008). *Tourism Management*, *33*(2), 361–370. |
| Both | Misener, L. (2015). Leveraging parasport events for community participation: Development of a theoretical framework. *European Sport Management Quarterly*, *15*(1), 132–153. |
| Both | Misener, L., Taks, M., Chalip, L., & Green, B. C. (2015). The elusive “trickle-down effect” of sport events: Assumptions and missed opportunities. *Managing Sport and Leisure*, *20*(2), 135–156 |
| Both | Montoya, C., Landolfi, E., Winkelman, M., Chamberlain, S. P., Fisch, K., & Wright, M. D. (2013). Olympic year impact on leisure-time physical activity rates within and across Canadian provinces and territories. *Physical Culture*, *67*(2), 143–147. |
| Both | Murphy, N. M., & Bauman, A. (2007). Mass sporting and physical activity events—Are they “Bread and Circuses” or public health interventions to increase population levels of physical activity? *Journal of Physical Activity & Health*, *4*(2), 193–202. |
| Both | Murphy, N., Lane, A., & Bauman, A. (2015). Leveraging mass participation events for sustainable health legacy. *Leisure Studies*, *34*(6), 758–766. |
| Both | Pappous, A., & Hayday, E. J. (2016). A case study investigating the impact of the London 2012 Olympic and Paralympic Games on participation in two non-traditional English sports, Judo and Fencing. *Leisure Studies*, *35*(5), 668–684. |
| Both | Piper, H., & Garratt, D. (2013). Olympic dreams and social realities: Legacy and mass participation. *Sociological Research Online*, *18*(2), 20. |
| Both | Potwarka, L. R., Drewery, D., Snelgrove, R., Havitz, M. E., & Mair, H. (2017). Modeling a demonstration effect: The case of spectators’ experiences at 2015 Pan Am Games’ track cycling competitions. *Leisure Sciences*, 1–23. |
| Both | Potwarka, L. R., & Leatherdale, S. T. (2016). The Vancouver 2010 Olympics and leisure-time physical activity rates among youth in Canada: Any evidence of a trickle-down effect? *Leisure Studies*, *35*(2), 241–257. |
| Both | Ramchandani, G. M., & Coleman, R. J. (2012). The inspirational effects of three major sport events. *International Journal of Event and Festival Management; Bingley*, *3*(3), 257–271. |
| Both | Ramchandani, G., Coleman, R. J., & Bingham, J. (2017). Sport participation behaviours of spectators attending major sports events and event induced attitudinal changes towards sport. *International Journal of Event and Festival Management, 8*(2), 121-135. |
| Both | Ramchandani, G., Davies, L. E., Coleman, R., Shibli, S., & Bingham, J. (2015). Limited or lasting legacy? The effect of non-mega sport event attendance on participation. *European Sport Management Quarterly*, *15*(1), 93–110. |
| Both | Ramchandani, G., Kokolakakis, T., & Coleman, R. (2014). Factors influencing the inspirational effect of major sports events on audience sport participation behaviour. *World Leisure Journal*, *56*(3), 220–235. |
| Both | Reis, A. C., de Sousa-Mast, F. R., & Gurgel, L. A. (2014). Rio 2016 and the sport participation legacies. *Leisure Studies*, *33*(5), 437–453. |
| Both | Rogerson, R. J. (2016). Re-defining temporal notions of event legacy: Lessons from Glasgow’s Commonwealth Games. *Annals of Leisure Research*, *19*(4), 497–518. |
| Both | Sandercock, G. R. H., Beedie, C., & Mann, S. (2016). Is Olympic inspiration associated with fitness and physical activity in English schoolchildren? A repeated cross-sectional comparison before and 18 months after London 2012. *BMJ Open*, *6*(11), e011670. |
| Both | Selvanayagam, M., Thompson, C., Taylor, S. J. C., Cummins, S., & Bourke, L. (2012). How might the London 2012 Olympics influence health and the determinants of health? Local newspaper analysis of pre-Games pathways and impacts. *BMJ Open*, *2*(6), e001791. |
| Both | Shipway, R. (2007). Sustainable legacies for the 2012 Olympic Games. *The Journal of the Royal Society for the Promotion of Health*, *127*(3), 119–124. |
| Both | Sousa-Mast, F. R., Reis, A. C., Gurgel, L. A., & Duarte, A. (2013). Are cariocas getting ready for the Games? Sport participation and the Rio de Janeiro 2016 Olympic Games. *Managing Leisure*, *18*(4), 331–335. |
| Both | Strittmatter, A.M., & Skille, E. Å. (2017). Boosting youth sport? Implementation of Norwegian youth sport policy through the 2016 Lillehammer Winter Youth Olympic Games. *Sport in Society*, *20*(1), 144–160. |
| Both | Such, E. (2016). The Olympic family? Young people, family practices and the London 2012 Olympic Games. *International Journal of Sport Policy and Politics*, *8*(2), 189–206. |
| Both | Taks, M., Green, B. C., Misener, L., & Chalip, L. (2014). Evaluating sport development outcomes: The case of a medium-sized international sport event. *European Sport Management Quarterly*, *14*(3), 213–237. |
| Both | Toohey, K. (2010). Post-Sydney 2000 Australia: A potential clash of aspirations between recreational and elite sport. *The International Journal of the History of Sport*, *27*(16–18), 2766–2779. |
| Both | Veal, A. J., Toohey, K., & Frawley, S. (2012). The sport participation legacy of the Sydney 2000 Olympic Games and other international sporting events hosted in Australia. *Journal of Policy Research in Tourism, Leisure and Events*, *4*(2), 155–184. |
| Both | Wang, W., & Theodoraki, E. (2007). Mass sport policy development in the Olympic City: The case of Qingdao — host to the 2008 sailing regatta. *The Journal of the Royal Society for the Promotion of Health*, *127*(3), 125–132. |
| Both | Weed, M., Coren, E., Fiore, J., Wellard, I., Mansfield, L., Chatziefstathiou, D., & Dowse, S. (2012). Developing a physical activity legacy from the London 2012 Olympic and Paralympic Games: A policy-led systematic review. *Perspectives in Public Health*, *132*(2), 75–80. |
| Both | Weed, M., Coren, E., Fiore, J., Wellard, I., Chatziefstathiou, D., Mansfield, L., & Dowse, S. (2015). The Olympic Games and raising sport participation: A systematic review of evidence and an interrogation of policy for a demonstration effect. *European Sport Management Quarterly*, *15*(2), 195–226. |
| Database | Aizawa, K., Wu, J., Inoue, Y., & Sato, M. (2018). Long-term impact of the Tokyo 1964 Olympic Games on sport participation: A cohort analysis. *Sport Management Review*, *21*(1), 86–97. |
| Database | Bason, T., & Grix, J. (2018). Planning to fail? Leveraging the Olympic bid. *Marketing Intelligence & Planning*, *36*(1), 138–151. |
| Database | Bauman, A, Murphy, N., & Lane, A. (2008). The role of community programmes and mass events in promoting physical activity to patients. *British Journal of Sports Medicine*, *43*(1), 44–46. |
| Database | Black, A., Costello, R., Craft, A., & Katene, W. (2015). ‘It’s all about developing the whole child’: An examination of the ‘legacy’ benefits of Youth Sport Trust’s school-based inclusion initiatives. *European Physical Education Review*, *21*(3), 362–378. |
| Database | Blauwet, C., & Willick, S. E. (2012). The Paralympic movement: Using sports to promote health, disability rights, and social integration for athletes with disabilities. *Pm&r*, *4*(11), 851–856. |
| Database | Clark, J., & Kearns, A. (2015). Pathways to a physical activity legacy: Assessing the regeneration potential of multi-sport events using a prospective approach. *Local Economy*, *30*(8), 888–909. |
| Database | Coaffee, J. (2013). Policy transfer, regeneration legacy and the summer Olympic Games: Lessons for London 2012 and beyond. *International Journal of Sport Policy and Politics*, *5*(2), 295–311. |
| Database | Coates, J., & Vickerman, P. B. (2016). Paralympic legacy: Exploring the impact of the Games on the perceptions of young people with disabilities. *Adapted Physical Activity Quarterly*, *33*(4), 338–357. |
| Database | Coleman, S. J., & Sebire, S. J. (2017). Do people’s goals for mass participation sporting events matter? A self-determination theory perspective. *Journal of Public Health*, *39*(4), E202–E208. |
| Database | Crofts, C., Dickson, G., Schofield, G., & Funk, D. (2012). Post-event behavioural intentions of participants in a women-only mass participation sporting event. *International Journal of Sport Management and Marketing, 12*(3-4), 260-274. |
| Database | Darko, N., & Mackintosh, C. (2016). ‘Don’t you feel bad watching the Olympics, watching us?’ A qualitative analysis of London 2012 Olympics influence on family sports participation and physical activity. *Qualitative Research in Sport, Exercise and Health*, *8*(1), 45–60. |
| Database | Downward, P., Dawson, P., & Mills, T. C. (2016). Sports participation as an investment in (subjective) health: A time series analysis of the life course. *Journal of Public Health*, *38*(4), E504–E510. |
| Database | Foley, M., McGillivray, D., & McPherson, G. (2012). Policy pragmatism: Qatar and the global events circuit. *International Journal of Event and Festival Management*, *3*(1), 101–115. |
| Database | Frawley, S., & Van den Hoven, P. (2015). Football participation legacy and Australia’s qualification for the 2006 Football World Cup. *Soccer & Society*, *16*(4), 482–492. |
| Database | Grix, J., Brannagan, P. M., Wood, H., & Wynne, C. (2017). State strategies for leveraging sports mega-events: Unpacking the concept of ‘legacy.’ *International Journal of Sport Policy and Politics*, *9*(2), 203–218. |
| Database | Hikoji, K., Chogahara, M., Tani, M., Sonoda, D., Matsumura, Y., Okada, A., ... & Ishizawa, N. (2012). The multidimensional benefits of participation in masters sports: A case study of the “Masters Koshien”. *International Journal of Sport and Health Science,* 201215. |
| Database | Hoskyn, K., Dickson, G., & Sotiriadou, P. (2018). Leveraging medium-sized sport events to attract club participants. *Marketing Intelligence & Planning*, *36*(2), 199–212. |
| Database | Inoue, Y., Berg, B. K., & Chelladurai, P. (2015). Spectator sport and population health: A scoping study. *Journal of Sport Management*, *29*(6), 705–725. |
| Database | Kaplanidou, K., & Gibson, H. J. (2010). Predicting behavioral intentions of active event sport tourists: The case of a small-scale recurring sports event. *Journal of Sport & Tourism*, *15*(2), 163–179. |
| Database | Kavetsos, G., & Szymanski, S. (2009). From the Olympics to the grassroots: What will London 2012 mean for sport funding and participation in Britain? *Public Policy Research*, *16*(3), 192–196. |
| Database | Koosha, M., Yoosefy, B., & Koosha, M. (2017). Understanding major sport events leveraging as social development: Future prospect of Iran. *Choregia*, *13*(1), 51–67. |
| Database | Liu, D., Broom, D., & Wilson, R. (2014). Legacy of the Beijing Olympic Games: A non-host city perspective. *European Sport Management Quarterly*, *14*(5), 485–502. |
| Database | Luciani, A., White, L., Berry, T. R., Deshpande, S., Latimer-Cheung, A. E., O’Reilly, N., … Faulkner, G. (2017). Sports Day in Canada: Examining the benefits for event organizers (2010-2013). *International Journal of Health Promotion and Education*, *55*(2), 66–80. |
| Database | Mackintosh, C., Darko, N., & May-Wilkins, H. (2016). Unintended outcomes of the London 2012 Olympic Games: Local voices of resistance and the challenge for sport participation leverage in England. *Leisure Studies*, *35*(4), 454–469. |
| Database | Müther, M., Williamson, M., & Williamson, L. (2014). Impact of the 2012 London Olympic and Paralympic Games on physical activity of Rheumatology patients. *JCR: Journal of Clinical Rheumatology, 20*(7), 376-378. |
| Database | Nightingale, C. M., Ram, B., Limb, E. S., Shankar, A., Procter, D., Cooper, A. R., … Owen, C. G. (2018). OP79 The effect of moving to east village (the former London 2012 Olympic Games athletes village) on physical activity and adiposity levels. *J Epidemiol Community Health*, *72*(Suppl 1), A38–A38. |
| Database | Perks, T. (2015). Exploring an Olympic “legacy”: Sport participation in Canada before and after the 2010 Vancouver Winter Olympics. *Canadian Review of Sociology/Revue Canadienne de Sociologie*, *52*(4), 462–474. |
| Database | Ram, B., Nightingale, C. M., Hudda, M. T., Kapetanakis, V. V., Ellaway, A., Cooper, A. R., … Owen, C. G. (2016). Cohort profile: Examining neighbourhood activities in built living environments in London: The ENABLE London—Olympic Park cohort. *Bmj Open*, *6*(10), e012643. |
| Database | Rauter, S. (2014). Mass sports events as a way of life (differences between the participants in a cycling and a running event). *Kinesiologia Slovenica*, *20*(1), 5–15. |
| Database | Reis, A. C., Sousa-Mast, F. R., & Vieira, M. C. (2014). Public policies and sports in marginalised communities: The case of Cidade de Deus, Rio de Janeiro, Brazil. *World leisure journal, 55*(3), 229-251. |
| Database | Sato, M., Jordan, J. S., & Funk, D. C. (2016). A distance-running event and life satisfaction: The mediating roles of involvement. *Sport Management Review*, *19*(5), 536–549. |
| Database | Silvey, D., Buote, R., Donovan, C., & Dubrowski, A. (2018). Impacts of the 2018 Newfoundland and Labrador Winter Games on youth who participated in the sport of Olympic Wrestling with Team Indigenous. *Physical & Health Education Journal; Gloucester*, *84*(2), 1–20. |
| Database | Smith, N. R., Clark, C., Fahy, A. E., Tharmaratnam, V., Lewis, D. J., Thompson, C., … Cummins, S. (2012). The Olympic Regeneration in East London (ORiEL) study: Protocol for a prospective controlled quasi-experiment to evaluate the impact of urban regeneration on young people and their families. *Bmj Open*, *2*(4), e001840. |
| Database | Stevinson, C., & Hickson, M. (2014). Exploring the public health potential of a mass community participation event. *Journal of Public Health*, *36*(2), 268–274. |
| Database | Strittmatter, A. M. (2016). Defining a problem to fit the solution: A neo-institutional explanation for legitimising the bid for the 2016 Lillehammer winter Youth Olympic Games. *International Journal of Sport Policy and Politics*, *8*(3), 421–437. |
| Database | Taks, M., Green, B. C., Misener, L., & Chalip, L. (2018). Sport participation from sport events: Why it doesn’t happen? *Marketing Intelligence & Planning*, *26*(2), 185–198. |
| Database | Taks, M., Chalip, L., & Green, B. C. (2015). Impacts and strategic outcomes from non-mega sport events for local communities. *European Sport Management Quarterly*, *15*(1), 1–6. |
| Database | Tan, T. C. (2015). The transformation of China’s national fitness policy: From a major sports country to a world sports power. *International Journal of the History of Sport*, *32*(8), 1071–1084. |
| Database | Wegman, O. (2018). Educational Olympic legacy: The public use of sport facilities after the Games.In V. Chis & I. Albulescu (Eds.), *Erd 2017—Education, Reflection, Development, Fifth Edition* (Vol. 41, pp. 884–889). |
| Database | Wegman, O. (2016). *Educational Olympic Challenge: The Legacy of Public Sport Participation*. 528–532. |
| Database | Wellings, K., Datta, J., Wilkinson, P., & Petticrew, M. (2011). The 2012 Olympics: Assessing the public health effect. *The Lancet*, *378*(9797), 1193–1195. |
| Database | White, L., Luciani, A., Berry, T. R., Deshpande, S., Latimer-Cheung, A., O’Reilly, N., … Faulkner, G. (2016). Sports day in Canada: A longitudinal evaluation. *International Journal of Health Promotion and Education*, *54*(1), 12–23. |
| Database | Zawadzki, K. M. (2016). Public perception of intangible benefits and costs in the valuation of mega sports events: The case of Euro 2012 in Poland. *Eastern European Economics*, *54*(5), 437–458. |
| Journal | Bell, B., & Daniels, J. (2018). Sport development in challenging times: Leverage of sport events for legacy in disadvantaged communities. *Managing Sport and Leisure*, *23*(4–6), 369–390. |
| Journal | Bloyce, D., & Lovett, E. (2012). Planning for the London 2012 Olympic and Paralympic legacy: A figurational analysis. *International Journal of Sport Policy and Politics*, *4*(3), 361–377. |
| Journal | Bowles, H. R., Rissel, C., & Bauman, A. (2006). Mass community cycling events: Who participates and is their behaviour influenced by participation? *International Journal of Behavioral Nutrition and Physical Activity*, 7. |
| Journal | Coalter, F. (2004). London 2012: A sustainable sporting legacy? In A. Vigor & M. Mean (Eds.), *After the Goldrush: A sustainable Olympics for London* (p. 14). London, UK: ippra Demos. |
| Journal | Coalter, F. (2007). London Olympics 2012: `the catalyst that inspires people to lead more active lives’? *The Journal of the Royal Society for the Promotion of Health*, *127*(3), 109–110. |
| Journal | Downward, P. M., & Ralston, R. (2006). The sports development potential of sports event volunteering: Insights from the XVII Manchester Commonwealth Games. *European Sport Management Quarterly*, *6*(4), 333–351. |
| Journal | Dubnewick, M., Hopper, T., Spence, J. C., & McHugh, T.-L. F. (2018). “There’s a Cultural Pride Through Our Games”: Enhancing the sport experiences of Indigenous youth in Canada through participation in Traditional Games. *Journal of Sport and Social Issues*, *42*(4), 207–226. |
| Journal | Early, F., & Corcoran, P. (2013). How can mass participation physical activity events engage low-active people? A qualitative study. *Journal of Physical Activity & Health*, *10*(6), 900–909. |
| Journal | Frawley, S., Toohey, K., & Veal, A. J. (Eds.). (2013). Managing sport participation legacy at the Olympic Games. In *Managing the Olympics* (p. 18). London, UK: Palgrave Macmillan. |
| Journal | Girginov, V. (2016). Has the London 2012 Olympic Inspire programme inspired a generation? A realist view. *European Physical Education Review*, *22*(4), 490–505. |
| Journal | Girginov, V., & Hills, L. (2009). The political process of constructing a sustainable London Olympics sports development legacy. *International Journal of Sport Policy and Politics*, *1*(2), 161–181. |
| Journal | Homma, K., & Masumoto, N. (2013). A theoretical approach for the Olympic legacy study focusing on sustainable sport legacy. *The International Journal of the History of Sport*, *30*(12), 1455–1471. |
| Journal | Howe, P. D., & Silva, C. F. (2018). The fiddle of using the Paralympic Games as a vehicle for expanding [dis]ability sport participation. *Sport in Society*, *21*(1), 125–136. |
| Journal | Hughes, K. (2012). Mega sports events and the potential to create a legacy of increased sport participation in the host country. In R. Shipway & A. Fyall (Eds.), *International Sports Events: Impacts, Experiences and Identities* (pp. 42–54). New York, NY: Routledge. |
| Journal | Inoue, Y., Heffernan, C., Yamaguchi, T., & Filo, K. (2018). Social and charitable impacts of a charity-affiliated sport event: A mixed methods study. *Sport Management Review*, *21*(2), 202–218. |
| Journal | Kaplanidou, K. (2017). Health related attitudes and behaviours among Olympic host city residents from Atlanta, Sydney, Athens, and Beijing Olympic Games: Exploring potential legacies. *Event Management*, *21*, 159–174. |
| Journal | Lane, A., Murphy, N., Bauman, A., & Chey, T. (2012). Active for a day: Predictors of relapse among previously active mass event participants. *Journal of Physical Activity & Health*, *9*(1), 48–52. |
| Journal | Lane, A., Murphy, N., & Bauman, A. (2013). An effort to ‘leverage’ the effect of participation in a mass event on physical activity. *Health Promotion International*, *30*(3), 542–551. |
| Journal | McCartney, G., Palmer, S., Winterbottom, J., Jones, R., Kendall, R., & Booker, D. (2010). A health impact assessment of the 2014 Commonwealth Games in Glasgow. *Public Health*, *124*(8), 444–451. |
| Journal | McCartney, G., Thomas, S., Thomson, H., Scott, J., Hamilton, V., Hanlon, P., … Bond, L. (2010). The health and socioeconomic impacts of major multi-sport events: Systematic review (1978-2008). *BMJ*, *340*(may19 4), c2369–c2369. |
| Journal | Misener, L., McGillivray, D., McPherson, G., & Legg, D. (2015). Leveraging parasport events for sustainable community participation: The Glasgow 2014 Commonwealth Games. *Annals of Leisure Research*, *18*(4), 450–469. |
| Journal | Nowak, P. F. (2012). Mass sports and recreation events as effective instruments of health-oriented education. *Journal of Physical Education & Health - Social Perspective*, *2*(3), 31–37. |
| Journal | Orr, M., & Jarvis, N. (2018). Blinded by gold: Toronto sports community ignores negative legacies of 2015 Pan Am Games. *Event Management*, *22*, 367–378. |
| Journal | Pappous, A. (2011). Do the Olympic Games lead to a sustainable increase in grassroots sport participation? In J. Savery & K. Gilbert (Eds.), *Sustainability and Sport* (pp. 81–89). Chicago: Common Ground Publishing. |
| Journal | Postlethwaite, V., Kohe, G. Z., & Molnar, G. (2018). Inspiring a generation: An examination of stakeholder relations in the context of London 2012 Olympics and Paralympics educational programmes. *Managing Sport and Leisure*, *23*(4–6), 391–407. |
| Journal | Potwarka, L. R. (2015). Exploring physical activity intention as a response to the Vancouver Olympics: An application and extension of the theory of planned behaviour. *Event Management*, *19*, 73–92. |
| Journal | Potwarka, L. R., Tepylo, H., Fortune, D., & Mair, H. (2016). Launching off but falling fast: Experiences of becoming more physically active in response to the Vancouver 2010 Olympic Winter Games. *Event Management, 20*(3), 297-312. |
| Journal | Ramchandani, G., Coleman, R., & Christy, E. (2017). The sport participation legacy of major events in the UK. *Health promotion international, 34*(1), 82-94. |
| Journal | Reis, A., Frawley, S., Hodgetts, D., Thomson, A., & Hughes, K. (2017). Sport participation legacy and the Olympic Games: The case of Sydney 2000, London 2012, and Rio 2016. *Event Management*, *21*(2), 139–158. |
| Journal | Sotiriadou, K., Shilbury, D., & Quick, S. (2008). The attraction, retention/transition, and nurturing process of sport development: Some Australian evidence. *Journal of Sport Management*, *22*(3), 247–272. |
| Journal | Taks, M., Misener, L., Chalip, L., & Green, B. C. (2013). Leveraging sport events for participation. *Canadian Journal for Social Research, 3*(1), 12-23. |
| Journal | Toohey, K. (2008). The Sydney Olympics: Striving for legacies – Overcoming short-term disappointments and long-term deficiencies. *The International Journal of the History of Sport*, *25*(14), 1953–1971. |
| Journal | Wicker, P., & Sotiriadou, P. (2013). The trickle-down effect: What populations benefit from hosting major sport events? *International Journal of Event Management Research, 8*(2), 17. |
| Journal | Yazici, T., Kocak, S., & Altunsoz, I. H. (2017). Examining the effect of experiential marketing on behavioral intentions in a festival with a specific sport event. *European Sport Management Quarterly*, *17*(2), 171–192. |
| Journal | Zhou, R., Kaplanidou, K., Papadimitriou, D., Theodorakis, N. D., & Alexandris, K. (2018). Understanding the inspiration among active participants in sport events. *International Journal of Event and Festival Management*, *9*(3), 332–348. |
